# Supplementary material for: Interplay between Extracellular Matrix Stiffness and JAM-A Regulates Mechanical Load on ZO-1 and Tight Junction Assembly
Source: Cell Rep. 2020 Jul 21;32(3):107924. doi: 10.1016/j.celrep.2020.107924 (PMC7383227; doi:10.1016/j.celrep.2020.107924)
Supplement: Document S1. Figures S1–S4 [file mmc1.pdf]

Cell Reports, Volume *32*

Supplemental Information

Interplay between Extracellular Matrix Stiffness and JAM-A Regulates Mechanical Load on ZO-1 and Tight Junction Assembly

Alexis J. Haas, Ceniz Zihni, Artur Ruppel, Christian Hartmann, Klaus Ebnet, Masazumi Tada, Maria S. Balda, and Karl Matter

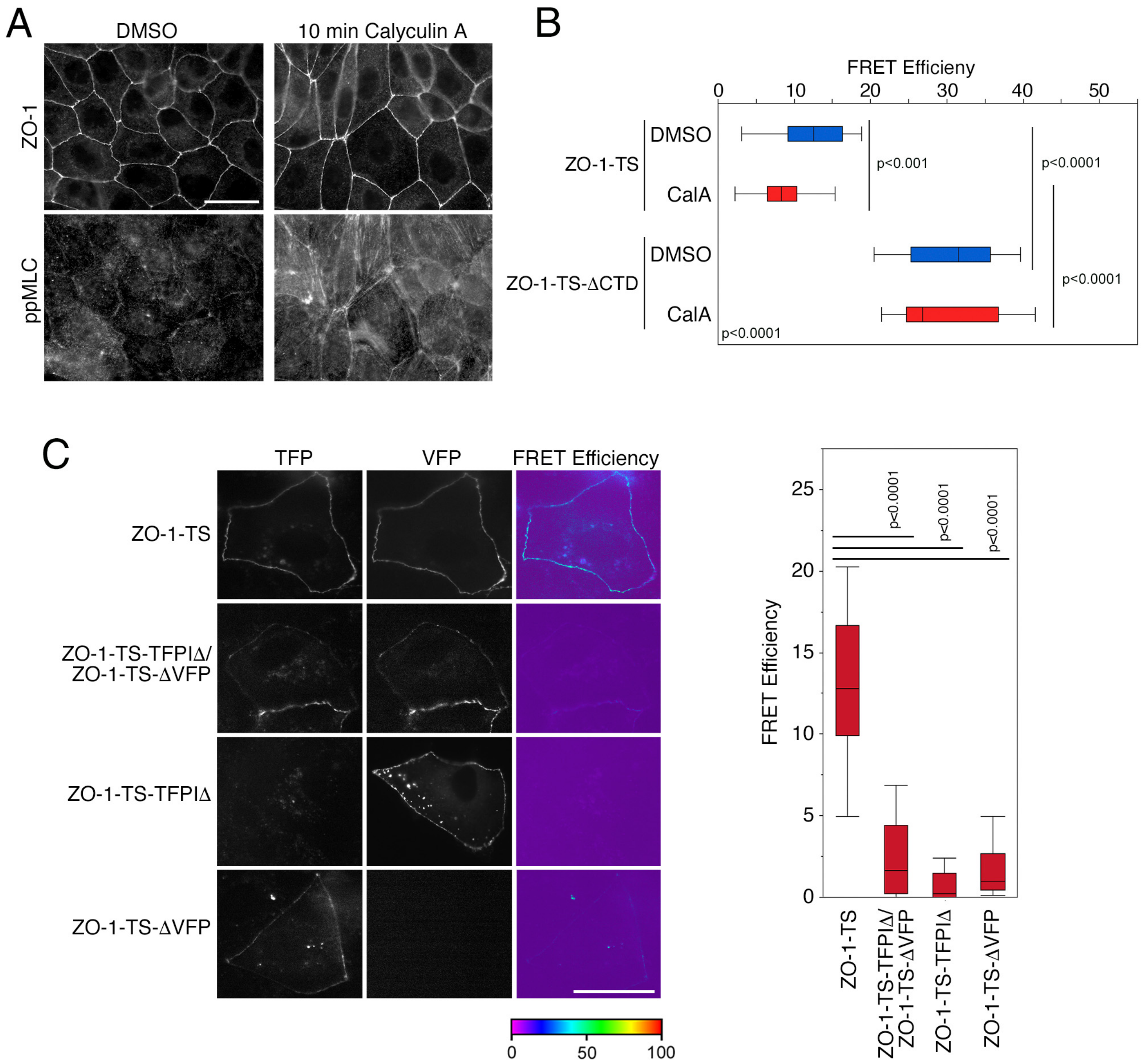

Figure S1: The ZO-1 sensor responds to increased cytoskeletal tension. Related to Figure 1.
(A) MDCK cells were treated with 20nM Calyculin A for 10 minutes prior to fixation and labelling for ZO-1 and double phosphorylated MLC. (B) FRET assay performed with cells expressing either ZO-1-TS or ZO-1-TS-ΔCTD after addition of vehicle or Calyculin A (images were taken between 10 and 20 minutes after addition; n for ZO-1-TS with DMSO, 34; ZO-1-TS-ΔCTD with DMSO, 17; ZO-1-TS with Calyculin A, 32; ZO-1-TS-ΔCTD with Calyculin A, 17; box-plot in panel E shows median and the interquartile ranges). (C) FRET assay performed with MDCK cells transfected with either ZO-1-TS, a ZO-1-TS variant with a deletion in the FRET donor TFP that renders it nonfluorescent (ZO-1-TS-TFPIΔ), a variant in which VFP had been deleted (ZO-1-TS-ΔVFP), or co-transfected with the two deletion variants (n for ZO-1-TS, 21; ZO-1-TS-TFPIΔ, 10; ZO-1-TS-ΔVFP, 21; ZO-1-TS-TFPIΔ/ZO-1-TS-ΔVFP, 20). Magnification bars, 20µm.

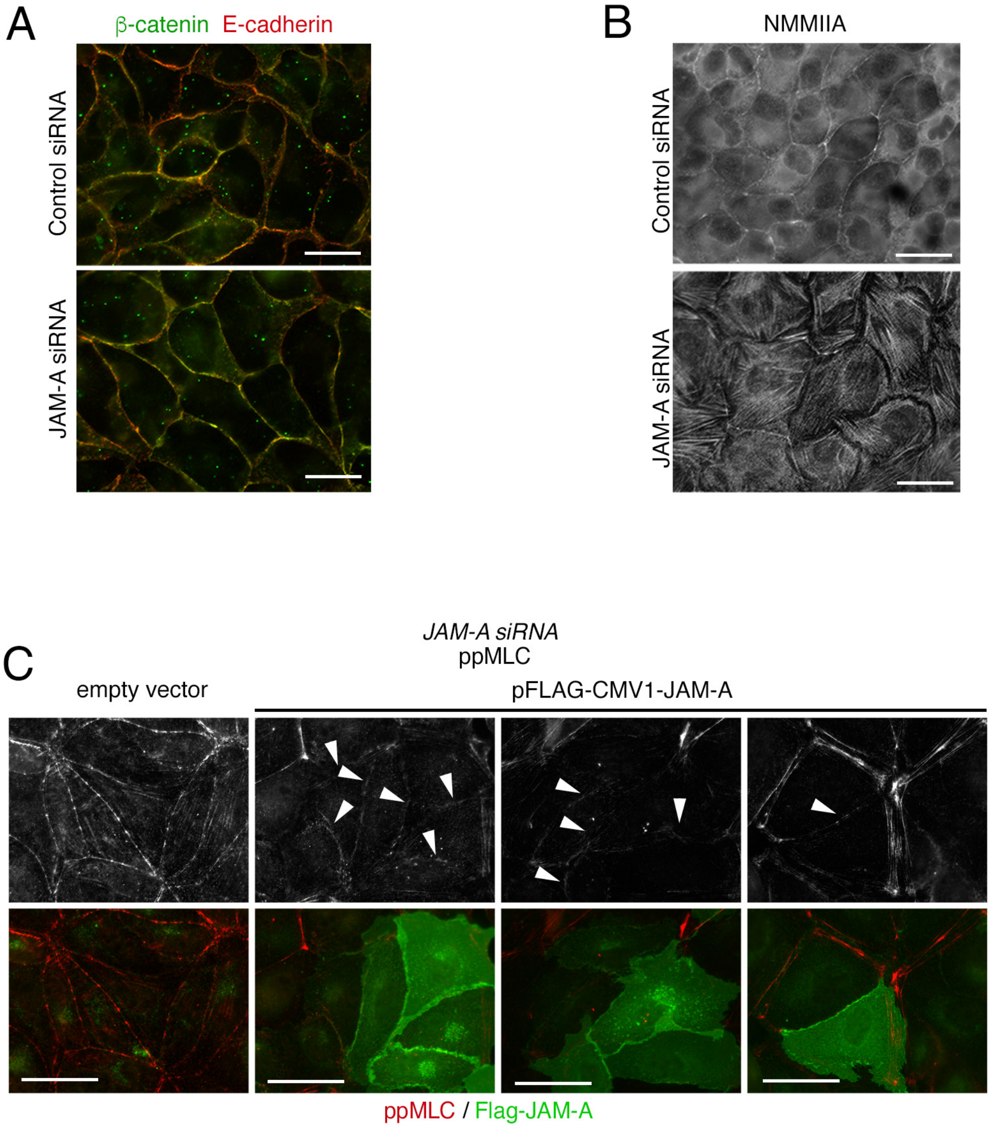

Figure S2: Effects and specificity of JAM-A depletion on MDCK cells. Related to Figure 2.
(A,B) MDCK cells were transfected with either control or JAM-A targeting siRNAs and were then fixed and stained for the adherens junction proteins E-cadherin and β-catenin (A) or the heavy chain of NMMIIA (B). (C) MDCK cells that had been transfected with JAM-A siRNAs were re-transfected with a plasmid encoding Flag-tagged human JAM-A. The cells were then stained for Flag-tagged JAM-A and double phosphorylated MLC. The first pair of images shows a field of cells transfected with an empty vector that are Flag-negative. Arrowheads label junction between cells that both re-express JAM-A and exhibit only low levels of ppMLC staining in contrast to junctions formed by cells that are JAM-A negative. Magnification bars, 20μm.

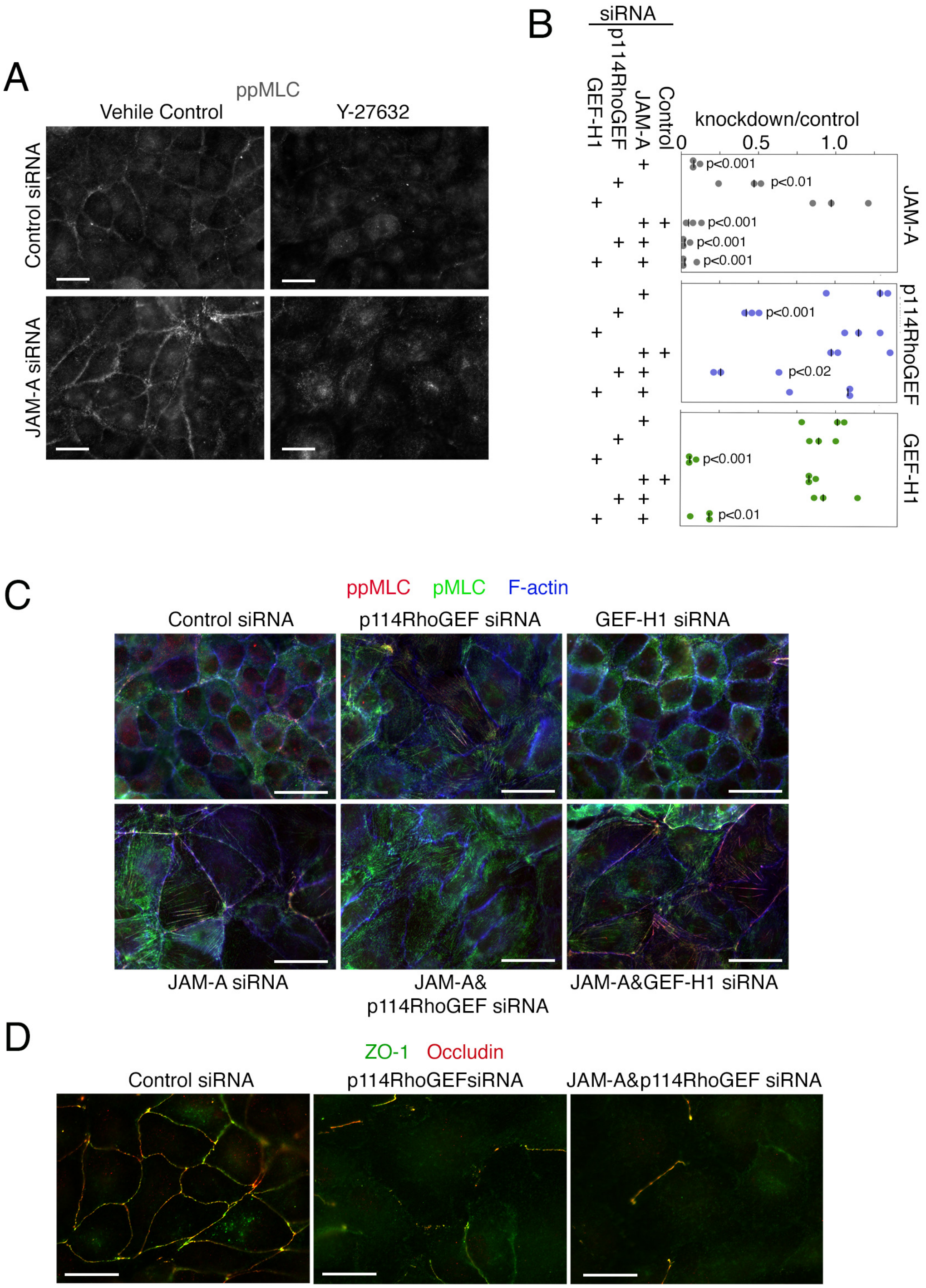

Figure S3: Analysis of signaling mechanisms downstream of JAM-A and importance of p114RhoGEF for tight junction integrity. Related to Figure 4.

(A) Control and JAM-A siRNA transfected cells were incubated with DMSO as a vehicle control or with the ROCKI/II inhibitor Y-27632 (10μM) for 5 hours prior to fixation and staining for ppMLC. (B) Quantification of immunoblots detecting JAM-A, p114RhoGEF and GEF-H1 expression. (C) Shown is an overlay of the ppMLC staining of Figure 4D with pMLC and F-actin of siRNA transfected MDCK cells. (D) MDCK cells transfected with control siRNA, p114RhoGEF siRNA, or JAM-A and p114RhoGEF siRNAs were fixed and processed for immunofluorescence using occludin and ZO-1 antibodies. Note, tight junction formation is p114RhoGEF-dependent in the presence or absence of JAM-A (cells only depleted of JAM-A are shown in Fig. 2C). Magnification bars, 20μm.

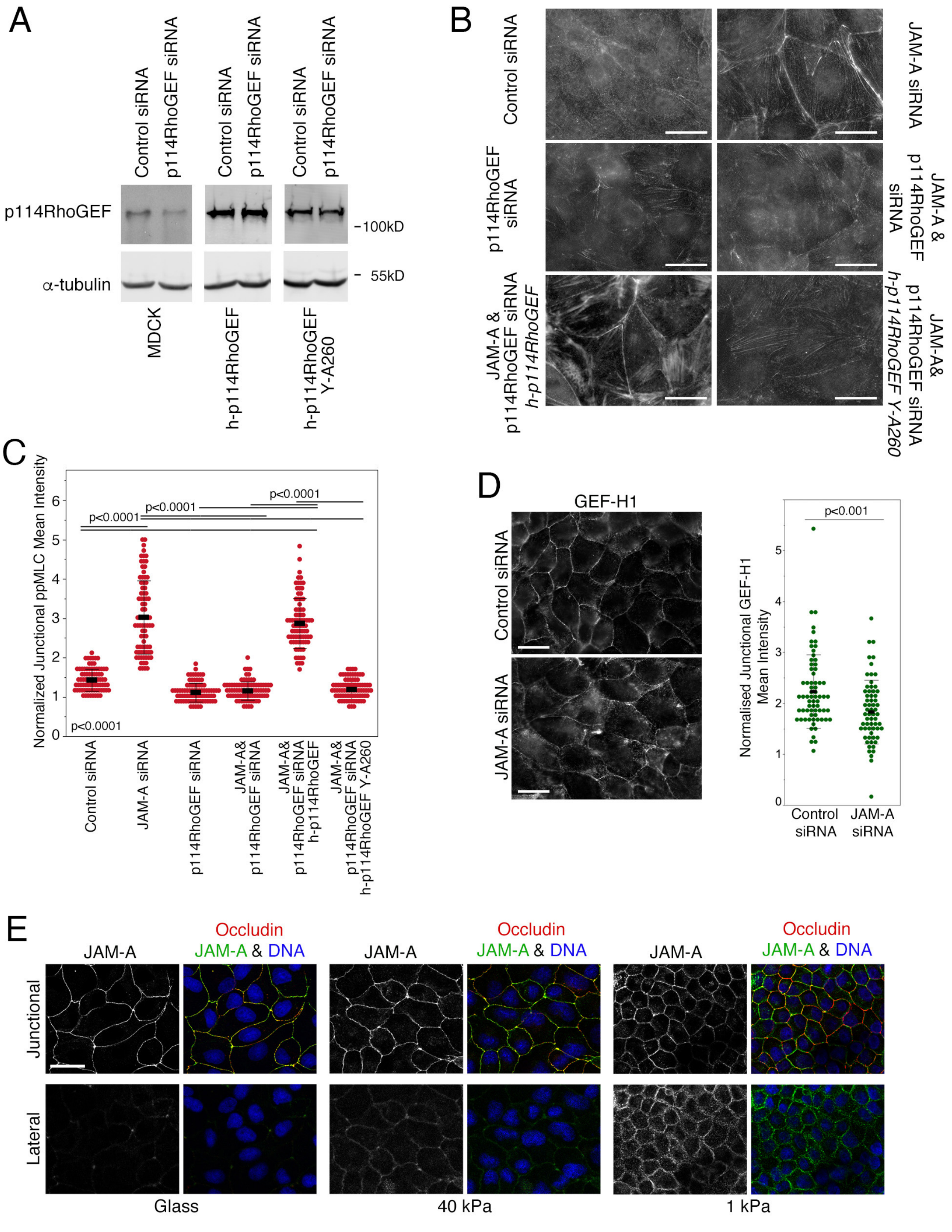

Figure S4: The catalytic activity of p114RhoGEF is required for junctional actomyosin activation in JAM-A depleted cells. Related to Figure 4.

(A-C) Rescue of junctional double phosphorylation of MLC in single or double siRNA-transfected MDCK cells was assayed after transfecting either active or inactive (Y-A260) human p114RhoGEF. Panel A shows an immunoblot demonstrating that human p114RhoGEF is not affected by the siRNAs that reduce expression of canine p114RhoGEF. Panel B shows immunofluorescence images of cells stained for double phosphorylated MLC. Panel C shows quantification of images from the experiment shown in panel B (81 cells were analyzed for each condition). (D) Control and JAM-A siRNA transfected MDCK cells were stained for GEF-H1. Junctional recruitment was quantified (n for control siRNA, 68; JAM-A siRNA, 63; shown are datapoints and means±1SD). (E) MDCK cells plated on Matrigel-coated glass coverslips or hydrogels were stained for JAM-A, occludin, and DNA to visualize nuclei. Shown are confocal sections taken at the junctional area and the lower half of the lateral membrane. Magnification bars, 20µm.
