# Supplementary material for: Interplay between Extracellular Matrix Stiffness and JAM-A Regulates Mechanical Load on ZO-1 and Tight Junction Assembly
Source: Cell Rep. 2020 Jul 21;32(3):107924. doi: 10.1016/j.celrep.2020.107924 (PMC7383227; doi:10.1016/j.celrep.2020.107924)
Supplement: Document S2. Article plus Supplemental Information [file mmc2.pdf]

Cell Reports

Report

Interplay between Extracellular Matrix Stiffness and JAM-A Regulates Mechanical Load on ZO-1 and Tight Junction Assembly

Graphical Abstract

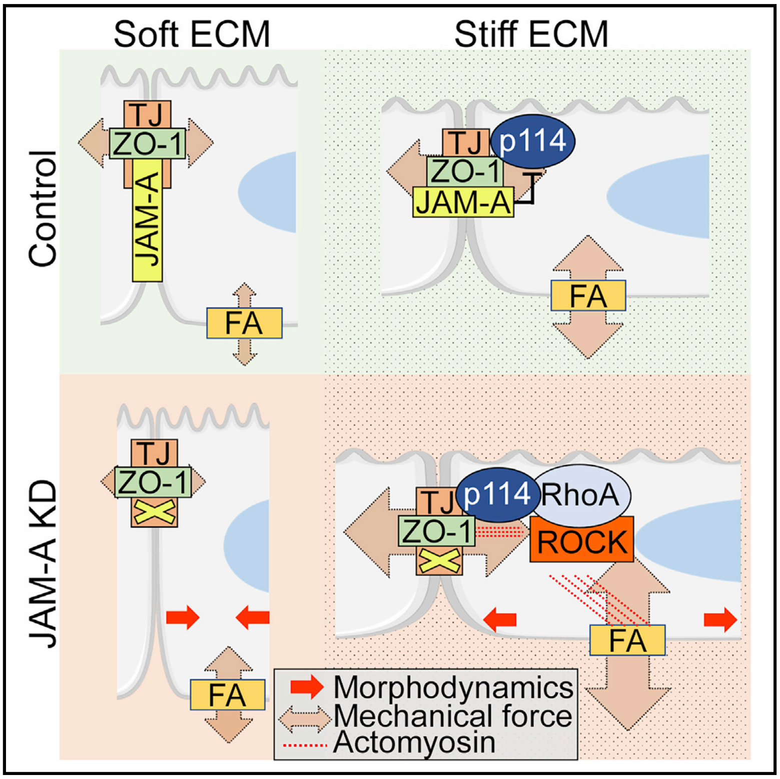

Highlights

- Tight junctions and ZO-1 bear a mechanical load
- ECM stiffness regulates mechanical tension on tight junctions and junction assembly
- JAM-A regulates mechanical forces at tight junctions and focal adhesions
- ECM-induced and junction-associated signaling cooperate to regulate junction assembly

Authors

Alexis J. Haas, Ceniz Zihni, Artur Ruppel, ..., Masazumi Tada, Maria S. Balda, Karl Matter

Correspondence

m.balda@ucl.ac.uk (M.S.B.), k.matter@ucl.ac.uk (K.M.)

In Brief

Haas et al. reveal that mechanical forces acting on tight junctions are regulated by extracellular matrix properties and a cell-cell adhesion molecule. The adhesion protein regulates the activation of a junctional actomyosin regulatory mechanism that is required for tight junction assembly when forces acting on the junction are high.

Haas et al., 2020, Cell Reports *32*, 107924
July 21, 2020 © 2020 The Author(s).
https://doi.org/10.1016/j.celrep.2020.107924

CellPress

CellPress
OPEN ACCESS

Report

Interplay between Extracellular Matrix Stiffness and JAM-A Regulates Mechanical Load on ZO-1 and Tight Junction Assembly

Alexis J. Haas,[1] Ceniz Zihni,[1] Artur Ruppel,[2] Christian Hartmann,[3] Klaus Ebnet,[3] Masazumi Tada,[4] Maria S. Balda,[1,*] and Karl Matter[1,5,*]
[1]UCL Institute of Ophthalmology, University College London, London EC1V 9EL, UK
[2]LiPhy, CNRS, Université Grenoble Alpes, Grenoble 38000, France
[3]Institute-associated Research Group "Cell adhesion and cell polarity," Institute of Medical Biochemistry, ZMBE, University of Münster, Münster 48149, Germany
[4]Department of Cell and Developmental Biology, University College London, London WC1E 6BT, UK
[5]Lead Contact
*Correspondence: m.balda@ucl.ac.uk (M.S.B.), k.matter@ucl.ac.uk (K.M.)
https://doi.org/10.1016/j.celrep.2020.107924

SUMMARY

Tight-junction-regulated actomyosin activity determines epithelial and endothelial tension on adherens junctions and drives morphogenetic processes; however, whether or not tight junctions themselves are under tensile stress is not clear. Here, we use a tension sensor based on ZO-1, a scaffolding protein that links the junctional membrane to the cytoskeleton, to determine if tight junctions carry a mechanical load. Our data indicate that ZO-1 is under mechanical tension and that forces acting on ZO-1 are regulated by extracellular matrix (ECM) stiffness and the junctional adhesion molecule JAM-A. JAM-A depletion stimulates junctional recruitment of p114RhoGEF/ARHGEF18, mechanical tension on ZO-1, and traction forces at focal adhesions. p114RhoGEF is required for activation of junctional actomyosin activity and tight junction integrity on stiff but not soft ECM. Thus, junctional ZO-1 bears a mechanical load, and junction assembly is regulated by interplay between the physical properties of the ECM and adhesion-regulated signaling at tight junctions.

INTRODUCTION

Intercellular junctions integrate mechanical forces during cell and tissue morphogenesis. The molecular composition of cell-cell junctions indicates that junctional complexes form molecular networks that link adhesion receptors to the cytoskeleton (Niessen and Gottardi, 2008). Hence, how intercellular junctions sense and transmit tension is a major research focus. The molecular linkages between adhesion receptors in adherens junctions and the actin cytoskeleton that transmit mechanical forces are well-known (Charras and Yap, 2018), but whether other cell-cell junctions, such as tight junctions, carry a mechanical load is not clear.

Tight junctions form paracellular diffusion barriers and function as signaling hubs that regulate epithelial and endothelial cell and tissue morphogenesis (Balda and Matter, 2016). They are formed by transmembrane adhesion, adaptor, and signaling proteins (Zihni et al., 2016). They regulate actomyosin activity and, thereby, cytoskeletal tension acting on adherens junctions (Cartagena-Rivera et al., 2017; Hatte et al., 2018; Tornavaca et al., 2015). Such regulatory processes are important for morphogenetic processes and epithelial and endothelial barrier properties.

Several tight junction components contain cytoskeletal binding sites; hence, they may also transmit cytoskeletal tension to the junction. ZO-1 is a scaffolding protein at the core of the junction. It interacts with transmembrane and cytosolic proteins by its N-terminal half (e.g., Claudins and JAMs) and possesses a C-terminal domain (CTD) with an actin-binding region (ABR; Figure 1A; Fanning and Anderson, 2009). Structural data and studies with purified proteins indicate that intramolecular interactions can mediate the formation of a closed form of ZO-1 unable to interact with ligands; stretching of ZO-1 has been proposed to be regulated by actomyosin activity (Lye et al., 2010; Spadaro et al., 2017). In wild-type cells, the apparent length of the junctional form is not sensitive to myosin inhibition, as it is stabilized by ZO-2 (Spadaro et al., 2017). Recently, ZO-1 was shown to form phase-separated cytosolic clusters that contain other cytosolic junctional proteins, such as ZO-2; phase separation requires ZO-1 domains that are not accessible in the closed conformation but does not require the ABR (Beutel et al., 2019; Schwayer et al., 2019). Phase separation is initiated in the cytosol, indicating that ZO-1 changes conformation prior to arrival at the junction and that the conformational switch is not powered by a junctional actomyosin-driven process. Hence, whether tight junctions are indeed a load-bearing structure has been questioned (Angulo-Urarte et al., 2020; Varadarajan et al., 2019).

Here, we show that junction-associated ZO-1 is under actomyosin-dependent tensile stress that is regulated by extracellular matrix (ECM) stiffness, indicating that the physical properties of the ECM impact on the mechanical load on ZO-1.

This is an open access article under the CC BY license (http://creativecommons.org/licenses/by/4.0/).

JAM-A, a cell-cell adhesion protein, is a negative regulator of cytoskeletal tension, and its junctional recruitment is regulated by ECM stiffness. Junctional actomyosin activation upon JAM-A depletion is stimulated by junctional recruitment of the RhoA activator p114RhoGEF, which is required for junction formation on stiff but not soft ECM, indicating that p114RhoGEF balances tensile forces generated at cell-ECM adhesions.

RESULTS

ZO-1 Is under Tensile Stress

Tensile forces acting on proteins can be measured by incorporating elastic modules into proteins that consist of fluorescent proteins functioning as FRET (fluorescence/Foerster resonance energy transfer) pairs that are linked by an elastic peptide (Grashoff et al., 2010). Increased tensile stress stretches the elastic module and thereby reduces the FRET efficiency. To test if ZO-1 is under tensile stress, we constructed a ZO-1-based sensor in which an elastic FRET module was inserted between the N-terminal motifs that interact with junctional partners and the CTD containing the ABR (Figure 1A). The sensor was efficiently expressed and recruited to tight junctions where it colocalized with occludin (Figures 1B and 1C). A construct containing the FRET module but lacking the CTD was generated as a negative control, as it cannot sense tensile stress. FRET experiments using a confocal microscope combined with an acceptor bleaching protocol revealed low FRET efficiency for the full-length ZO-1 sensor and a higher FRET efficiency for the ΔCTD construct, indicating that the ZO-1 sensor was under tensile stress (Figures 1D and 1E). An addition of Blebbistatin, a nonmuscle myosin-II (NMMII) inhibitor, led to increased FRET efficiency, indicating relaxation. The difference in FRET efficiency between control and Blebbistatin-treated samples corresponds to a force of approximately 2–3 pN (Grashoff et al., 2010). Thus, junctional ZO-1 is under actomyosin-dependent tensile stress.

The Physical Properties of the ECM Regulate the Mechanical Force on Junctional ZO-1

We next asked whether physiological modulation of cytoskeletal tension impacts on tension sensed by ZO-1. ECM stiffness regulates cytoskeletal tension through mechanotransduction at focal adhesions (Galbraith and Sheetz, 1998). Hence, we transfected cells on Matrigel-coated substrates of different stiffnesses with the ZO-1-based tension sensor and measured FRET efficiencies by using a filter-based epifluorescence system. FRET efficiencies increased with decreasing ECM stiffness (Figures 1F and 1G), indicating that tension acting on ZO-1 is regulated by the substrate stiffness.

We next stimulated cytoskeletal tension with calyculin A, a potent phosphatase inhibitor that stimulates cortical actomyosin contraction, to determine whether the sensor can detect increased tension (Acharya et al., 2018; Asano and Mabuchi, 2001; Henson et al., 2003). Short incubations with calyculin A led to enhanced myosin light chain (MLC) phosphorylation, indicating NMMII activation, and reduced FRET efficiency, indicating increased tension (Figures S1A and S1B).

Although the results indicate that the ZO-1 sensor responds to cytoskeletal tension, part of the FRET signal could stem from inter- rather than intramolecular energy transfer. Co-expression of two mutant sensors in which either one of the fluorescent proteins was inactivated did not reveal significant FRET activity (Figure S1C). Hence, the contribution of intermolecular FRET to the signal obtained with the ZO-1 tension sensor is negligible.

JAM-A Regulates Tensile Stress of ZO-1 and ECM Strain

JAM-A is an adhesion receptor, which directly interacts with the PDZ3 domain of ZO-1 (Ebnet et al., 2000). JAM-A has been suggested to stabilize the epithelial barrier function by limiting RhoA and NMMII activities by unknown mechanisms; hence, it may regulate tensile stress of ZO-1 (Monteiro et al., 2013). Depletion of JAM-A stimulated MLC phosphorylation, indicating NMMII activation (Figures 2A and 2B). Markers of tight (occludin and ZO-1) and adherens junctions (E-cadherin, p120catenin, and β-catenin) were still recruited to cell-cell junctions (Figure 2C; Figure S2A), indicating no general defects in junction assembly. Depletion of JAM-A also enhanced stress fiber and focal adhesion formation, which are signs of increased RhoA activation (Figures 2D and 2E; Figure S2B). Consequently, JAM-A-depleted cells were more spread than control cells on stiff ECM but not when plated on a soft 1-kPa matrix, which attenuates ECM-induced cytoskeletal tension (Figures 2F and 2G). On a 1-kPa ECM, JAM-A-depleted cells were slightly smaller than controls, suggesting that JAM-A depletion may increase the traction on the ECM and, hence, gel contraction. Increased phosphorylation of MLC was observed along stress fibers but also at cell-cell junctions and was induced by small interfering RNAs (siRNAs) targeting different sequences but rescued by the expression of siRNA-resistant, FLAG-tagged JAM-A, indicating that the induction of junctional NMMII activity was specific (Figure 2D; Figure S2C).

We next used the tension sensor to ask if JAM-A depletion affects tension on ZO-1. JAM-A depletion reduced FRET efficiency, indicating increased junctional tension (Figures 3A and 3B). A tension sensor based on the adherens junction adhesion protein E-cadherin (Borghi et al., 2012) only revealed an insignificant reduction in FRET efficiency upon JAM-A depletion. Thus, JAM-A negatively regulates tensile forces acting on ZO-1.

JAM-A depletion induced stress fiber formation and focal adhesion remodeling. Hence, we used traction force microscopy (TFM) to measure the stress on the ECM induced by the cells (Alkasalias et al., 2017; Butler et al., 2002; Milloud et al., 2017). Islands of cells plated on polyacrylamide (PAA) hydrogels (average stiffness of ∼16.4 kPa) and surrounded by an ample margin of empty matrix were imaged. Although this soft ECM led to a weaker phenotype than glass as expected, the cells still appeared more spread. JAM-A depletion increased traction forces on the ECM, as revealed by an increase in strain energy density (Figures 3C–3E). Thus, JAM-A depletion induced cell-wide changes in actomyosin organization that led to increased cytoskeletal tension on tight junctions and focal adhesions.

JAM-A Regulates p114RhoGEF Signaling

To identify the mechanism behind JAM-A regulation of junctional actomyosin activation, we used a FRET biosensor to determine the involvement of RhoA activation (Yoshizaki et al., 2003). Depletion of JAM-A increased FRET efficiency at cell-cell

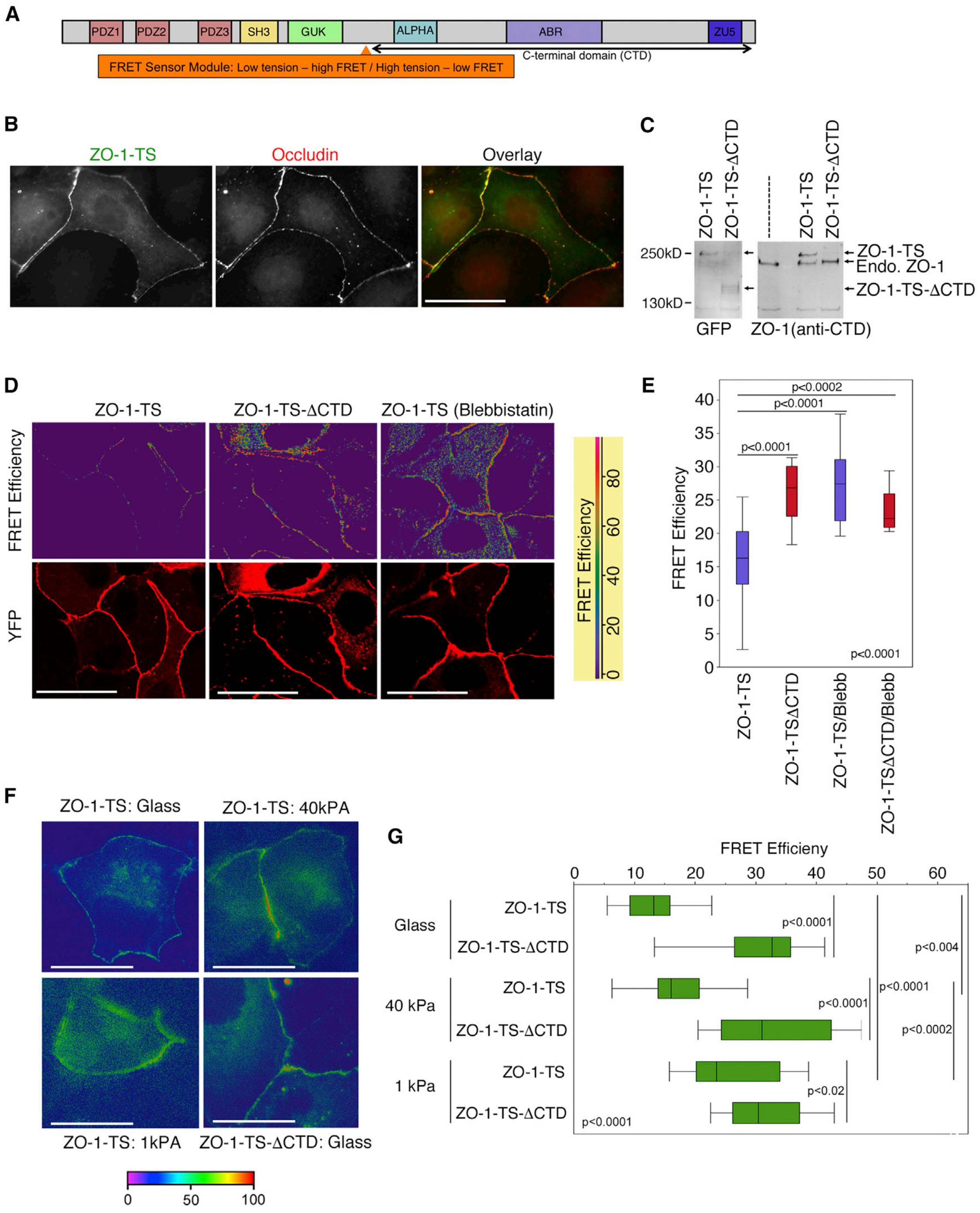

Figure 1. ZO-1 Is under Tensile Stress Regulated by Matrix Stiffness

(A) Domain structure of ZO-1. Indicated are the main structural domains and the insertion site of the FRET module.

(B and C) The ZO-1 tension sensor (ZO-1-TS) was transiently expressed in Madin-Darby canine kidney (MDCK) cells prior to an analysis of localization by immunofluorescence (B) and immunoblotting (C) with anti-GFP or anti-CTD antibodies.

(D and E) FRET analysis by acceptor bleaching and confocal microscopy of full-length ZO-1-TS and the control sensor lacking the CTD. Blebbistatin (10 μM) was added for 20 min prior to imaging. The yellow fluorescent protein (YFP) image taken prior to bleaching reveals the localization of the sensor. The graph shows a quantification of junctional FRET efficiencies of analyzed cells (n for ZO-1-TS, 23; ZO-1-TS-ΔCTD, 17; ZO-1-TS with Blebbistatin, 17; ZO-1-TS-ΔCTD with Blebbistatin, 11; box-plot shows median and interquartile ranges).

(F and G) FRET analysis by epifluorescence microscopy of control siRNA-transfected MDCK cells plated on Matrigel-coated glass coverslips or hydrogels of different stiffnesses prior to transfection of the ZO-1 sensor (F). (G) Shows FRET efficiencies at cell-cell contacts of n analyzed cells (n for ZO-1-TS glass, 31; 40 kPa, 31; 1 kPa, 20; ZO-1-TS-ΔCTD glass, 17; 40 kPa, 18; 1 kPa, 18; box-plot shows median and interquartile ranges). Magnification bars, 20 μm. See also Figure S1.

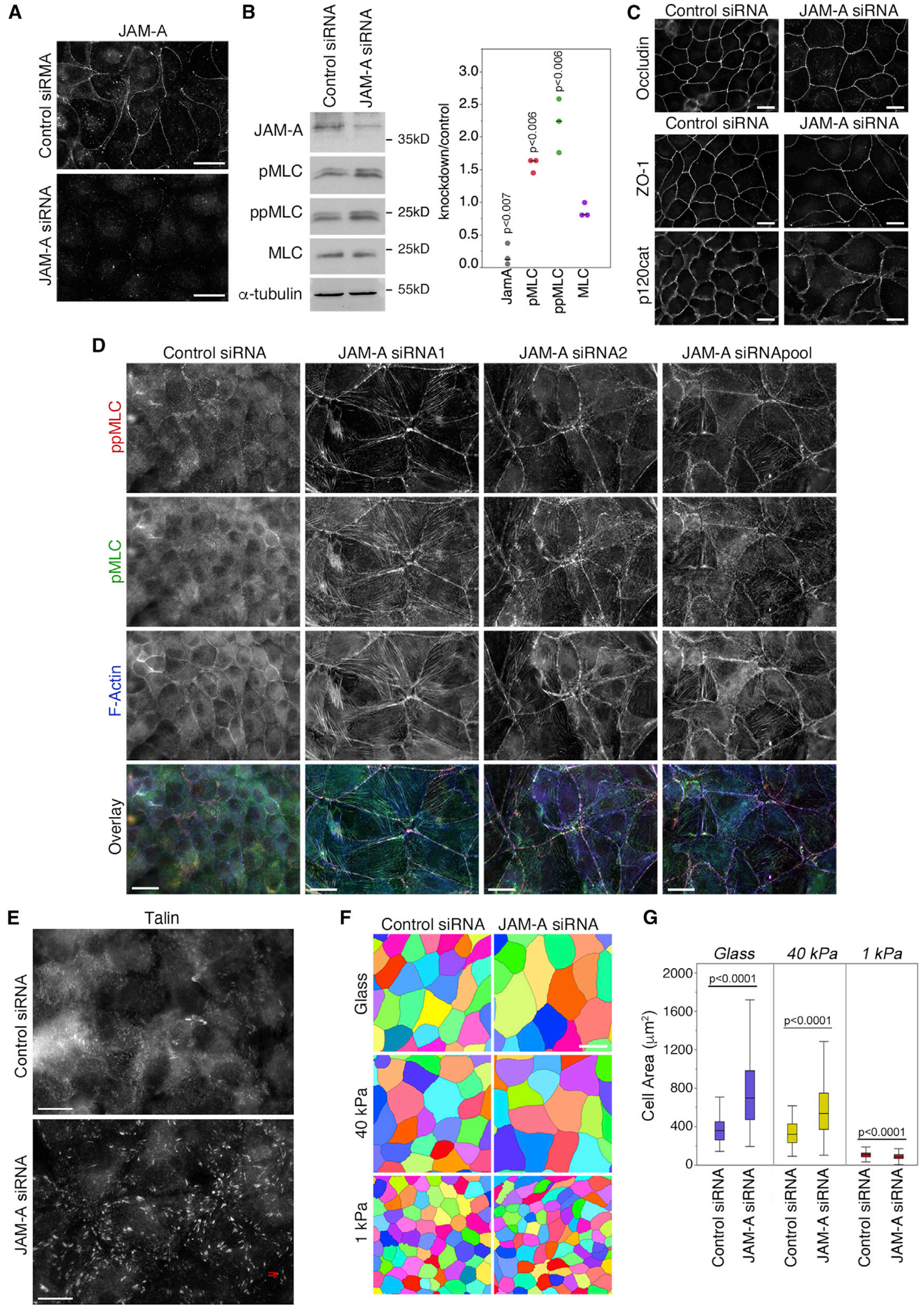

(legend on next page)

junctions (Figure 4A). This was paralleled by the stimulation of junctional recruitment of p114RhoGEF, an activator of RhoA (Figure 4B). p114RhoGEF drives RhoA signaling during junction formation and, when active, forms a stable complex with ROCKII, leading to a preferential increase of double-phosphorylated MLC (Terry et al., 2011); indeed, JAM-A-depletion-induced junctional MLC phosphorylation was blocked by a ROCKI/II inhibitor (Figure S3A). Double knockdown experiments revealed that depletion of p114RhoGEF attenuated junctional NMMII activation in JAM-A-depleted cells and disrupted tight junctions, indicating that junctional actomyosin regulation downstream of JAM-A required p114RhoGEF (Figures 4C–4E; Figures S3B–S3D). Junctional myosin activation was rescued by the expression of siRNA-resistant p114RhoGEF but not by a catalytically inactive mutant (Figures S4A–S4C). Depletion of p114RhoGEF also stimulated downregulation of JAM-A, suggesting a feedback loop between junctional RhoA signaling and JAM-A (Figure 4C; Figure S3B). Depletion of GEF-H1, a RhoA guanine nucleotide exchange factor (GEF) whose junctional localization was reduced upon JAM-A depletion, did not affect junctional MLC phosphorylation (Figure 4D-E; Figure S4D). Basal stress fiber formation and myosin activation were not affected by either knockdown of GEF-H1 or p114RhoGEF in JAM-A-depleted cells (Figure S3B). Thus, JAM-A regulates the junctional recruitment of the RhoA activator p114RhoGEF to stimulate junctional actomyosin activity.

As ECM stiffness impacts on tension on junctional ZO-1, we asked whether reduced ECM stiffness and, hence, less cytoskeletal tension, rescues junction formation in p114RhoGEF-depleted cells. Indeed, junction formation was affected most strongly on glass and less on 40-kPa ECM and was not affected by p114RhoGEF depletion on a very soft 1-kPa ECM (Figures 4F and 4G). Thus, p114RhoGEF supports tight junction formation by counteracting high basal tension, suggesting it is part of a junctional mechanosensing mechanism. JAM-A was only efficiently concentrated at tight junctions on stiff ECM, suggesting that junctional recruitment of the p114RhoGEF regulator is tension dependent (Figure S4E).

DISCUSSION

Our data identify ZO-1 as a tension-transmitting junctional adaptor, indicating that tight junctions bear a mechanical load and suggesting that ZO-1 is part of a force transmitting molecular bridge between the cytoskeleton and tight junctions (Figure 4H). Mechanical load is regulated by the physical properties of the ECM and the adhesion protein JAM-A, whose junctional recruitment is regulated by ECM stiffness. JAM-A depletion activates junctional actomyosin activity by stimulating p114RhoGEF recruitment, which is required for junction formation in cells on stiff ECM, suggesting that p114RhoGEF recruitment is part of a mechanosensing mechanism. Hence, ZO-1 not only transmits tensile forces but also forms a molecular platform for the recruitment of a force-sensing and -generating machinery that is activated in response to changes in cell-cell adhesion and cytoskeletal tension.

Depletion of JAM-A induced actomyosin remodeling and increased traction forces on the ECM. Thus, adhesion molecules of tight junctions signal to regulate the junctional cytoskeleton and cell-ECM adhesion. This crosstalk is bidirectional, as ECM stiffness also stimulated tensile stress of ZO-1. JAM-A depletion also induced apparent cell flattening, an effect that was counteracted by plating cells on a soft ECM. Hence, interplay between adhesion at tight junctions and to the ECM leads to cell-wide changes in actomyosin activity that determine cell morphology. Basal actomyosin activation associated with JAM-A depletion was not p114RhoGEF dependent. We also could not detect a role for GEF-H1, a GEF linked to RhoA activation, upon pulling on non-junctional JAM-A in endothelial cells (Scott et al., 2016). Hence, the mechanism stimulating basal contractility remains to be identified.

ZO-1 interacts with multiple transmembrane proteins (Zihni et al., 2016). The relevant adhesion receptors that transmit mechanical forces between neighboring cells are not known. Such receptors may function cooperatively, as individual junctional adhesion proteins appear to have low adhesive strength (Van Itallie and Anderson, 1997; Zihni et al., 2016). ZO proteins form a condensate that interacts with other cytosolic and transmembrane proteins; hence, individual adhesion proteins may not function independently, as they interact with a large phase-separated structure (Beutel et al., 2019). This idea is supported by a study showing that multiple transmembrane components need to be inactivated to prevent tight junction assembly (Otani et al., 2019). Hence, ZO-1 may be part of a mechanical force-transducing bridge to a group of adhesion proteins that form a multivalent adhesive unit.

Actomyosin activity has been linked to a conformational switch of ZO-1 that regulates the binding of ligands (Spadaro et al., 2017). The conformational switch only occurs when cells are depleted of ZO-2, a protein that interacts with ZO-1, and are treated with a myosin inhibitor. The actomyosin-regulated, force-sensitive stretching we observed does not correspond to the conformational switch as it occurs in the presence of ZO-2. *In vitro*, maintenance of a fully stretched form of ZO-1 requires a force of 2–4 pN (Spadaro et al., 2017). This is similar to the force required to stretch the ZO-1 tension sensor; hence, the fully

Figure 2. JAM-A Regulates Actomyosin Remodeling

(A and B) Depletion of JAM-A in MDCK cells was induced by transfection of siRNAs and was monitored by immunofluorescence (A) or immunoblotting (B).

(C) Control and JAM-A-depleted cells were stained for markers of tight (occludin and ZO-1) and adherens junctions (p120-catenin).

(D and E) MDCK cells were transfected with either control or JAM-A-targeting siRNAs before fixation and staining for double- and single-phosphorylated MLC to reveal active NMMII and F-actin (D) or talin to reveal focal adhesions (E).

(F and G) Cells transfected with siRNAs were plated on Matrigel-coated coverslips or 40 kPa or 1 kPa hydrogels before immunofluorescence. The apical surface area was then quantified as a measure for cell spreading by obtaining a cell segmentation based on ZO-1 staining (glass control siRNA, 81 cells; JAM-A siRNA, 55 cells; 40 kPa control siRNA, 102 cells; and JAM-A, 73 cells; 1 kPa control siRNA, 298 cells; and JAM-A, 287 cells; box-plot shows median and the interquartile range). Magnification bars, 20 μm. See also Figure S2.

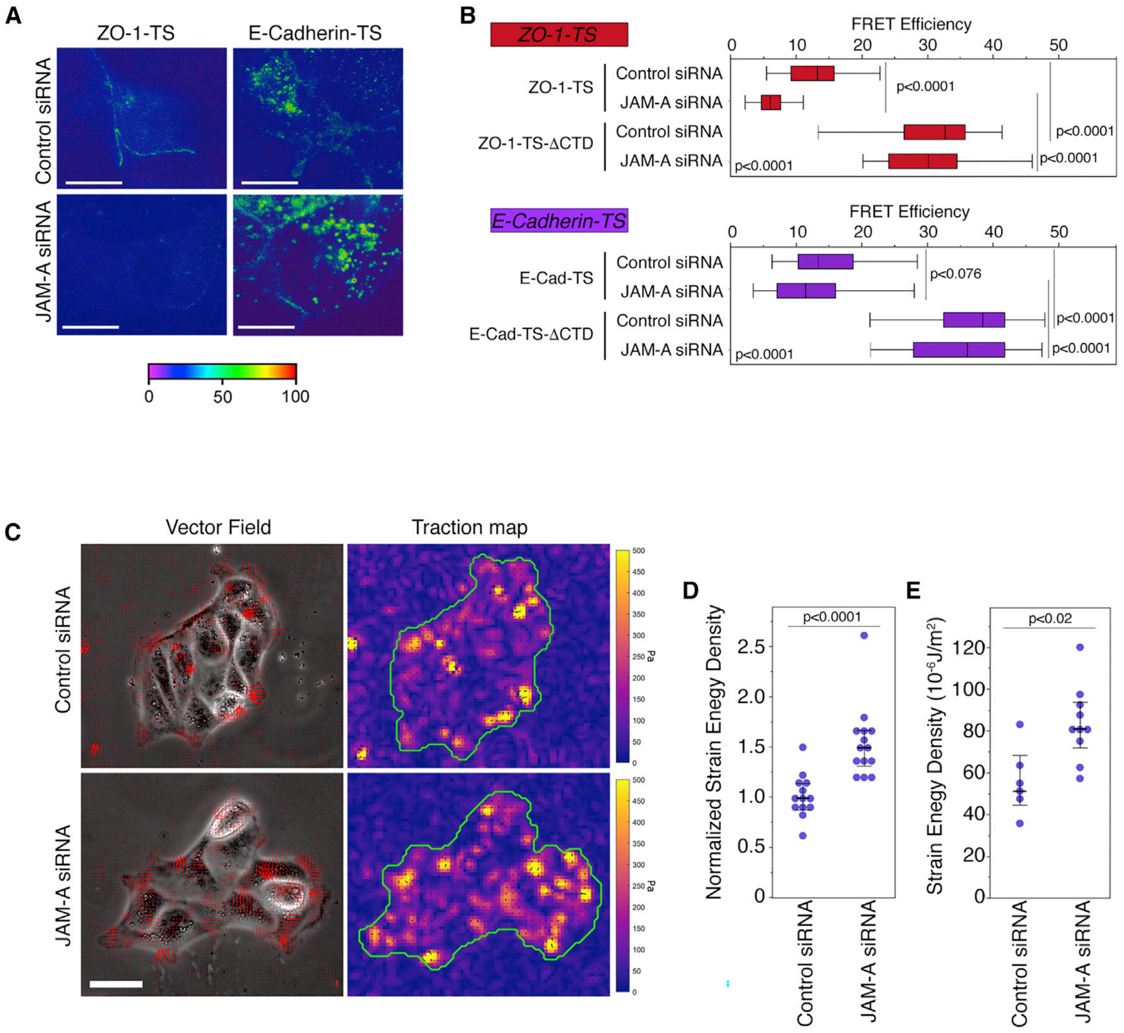

Figure 3. JAM-A Regulates Mechanical Stress on Tight Junctions and ECM

(A and B) FRET analysis by epifluorescence microscopy of MDCK cells transfected with siRNAs and ZO-1- or E-cadherin-based sensors. The quantification shows FRET efficiencies at cell-cell contacts (n for ZO-1-TS control siRNA, 31, and JAM-A siRNA, 37; ZO-1-TS-ΔCTD control siRNA, 17, and JAM-A siRNA, 20; E-cadherin-TS control siRNA, 34, and JAM-A siRNA, 31; and E-cadherin-TS-ΔCTD control siRNA, 19, and JAM-A siRNA, 19; ZO-1-TS and ZO-1-TS-ΔCTD control siRNA values are the same as those shown in Figure 1F, as the conditions were tested in parallel; box-plots show median and interquartile ranges).
(C–E) TFM on cells transfected with siRNAs as indicated and plated on PAA hydrogels. (C) shows images of the traction vector fields overlaid on phase contrast images and the corresponding stress maps on which the borders of the cell island is displayed. The quantifications in (D) and (E) show the derived strain energy density datapoints as the average value of all islands in single gels, and then show them normalized to respective controls to include 3 independent experiments (D) and absolute values of single islands of one representative experiment (medians with interquartile ranges are indicated; E). Magnification bars, 20 μm (A); 50 μm (C).

stretched form observed *in vitro* may represent ZO-1 under actomyosin-generated tension in tight junctions. Therefore, dimerization with ZO-2 during junction assembly may stabilize an open conformation, and in a second step, tension-induced stretching then relies on actomyosin-generated force. Although Spadaro et al. (2017) did not identify distinct stretched forms in cells using super-resolution microscopy, this might have been due to the resolution limits of structured illumination microscopy, as the stretched form measures only 75 nm and was detected in cells by indirect immunofluorescence labeling of both ends. Inhibition of myosin leads to ZO-1 folding only in the absence of ZO-2, as the latter is required to maintain the open conformation. Binding of internal ligands such as ZONAB is therefore only sensitive to myosin inhibition and cytoskeletal tension in the absence of ZO-2 but not in wild-type cells (Spadaro et al., 2017). ZONAB activation is indeed not directly related to actomyosin activity, as stimulation of RhoA by GEF-H1 activates ZONAB in a Rho-associated coiled-coil containing protein kinase (ROCK)-independent manner (Nie et al., 2009). ZO-1 undergoes phase separation and engages with ligands prior to arrival at the junctional membrane; hence, it is conceivable that unfolding of ZO-1 and subsequent ligand binding are regulated by components that

CellPress
OPEN ACCESS

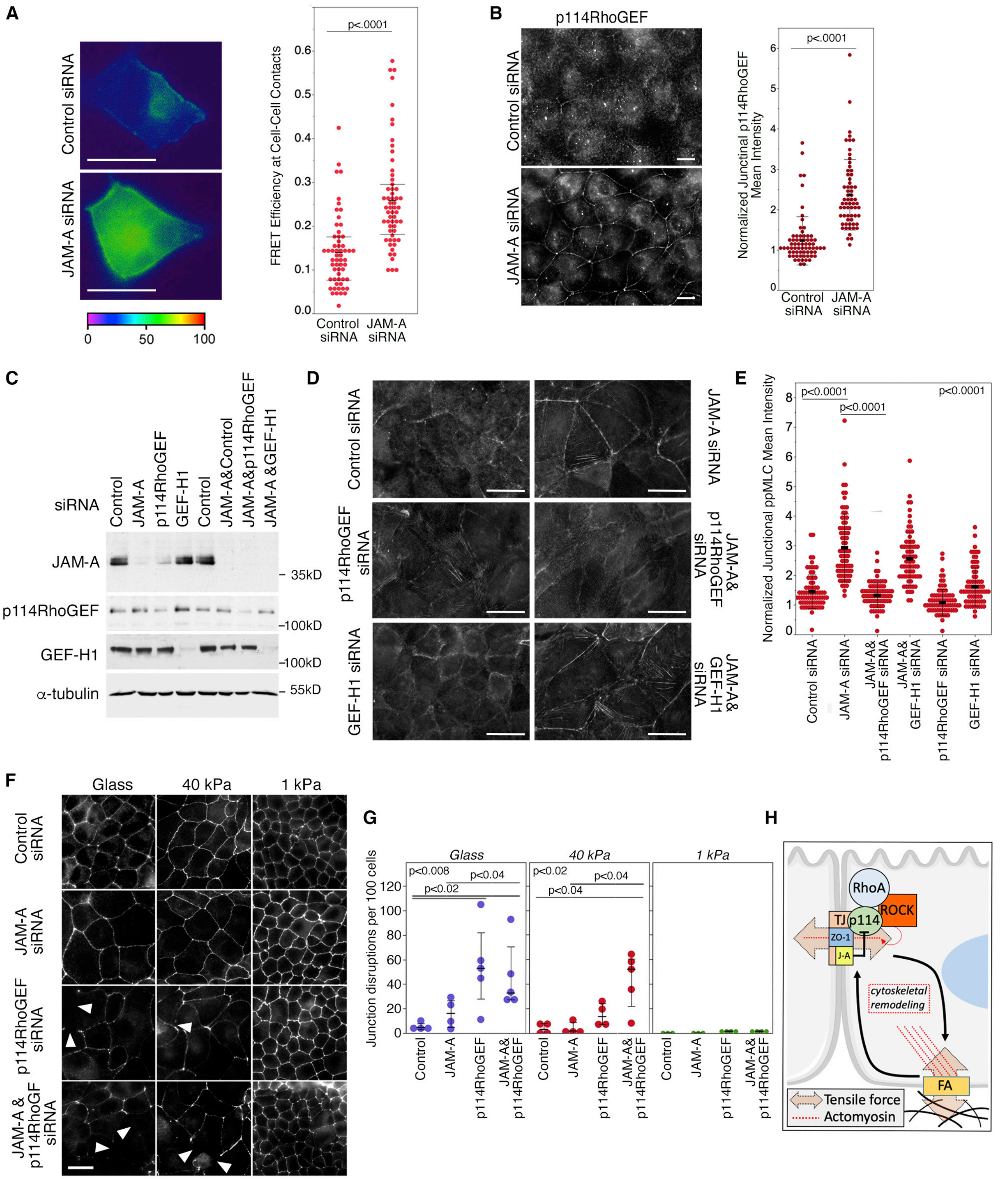

Figure 4. p114RhoGEF Regulates Junctional Actomyosin Remodeling and Tight Junction Assembly in JAM-A-Depleted Cells

(A) Control and JAM-A-depleted cells were transfected with a FRET biosensor for RhoA activation, and FRET efficiency at cell-cell contacts was quantified (n for both conditions was 58; shown are the data values along with the median and the interquartile range).

(B) Control and JAM-A-depleted cells were stained for p114RhoGEF, and junctional recruitment was quantified (n for control siRNA, 68; JAM-A siRNA, 63; shown are datapoints and means ± 1 SD).

(legend continued on next page)

only transiently associate with ZO-1, such as the ZO-1-binding heat shock protein Apg-2 (Aijaz et al., 2007; Tsapara et al., 2006). Hence, further investigations into the mechanisms that regulate the interplay between cytoskeletal tension and signaling at tight junctions will be key to our understanding of the role of tight junctions in morphogenetic processes and responses to tissue stress.

STAR★METHODS

Detailed methods are provided in the online version of this paper and include the following:

- KEY RESOURCES TABLE
- RESOURCE AVAILABILITY
  - Lead Contact
  - Materials Availability
  - Data and Code Availability
- EXPERIMENTAL MODEL AND SUBJECT DETAILS
- METHOD DETAILS
  - Small Molecule Inhibitors
  - Expression Plasmids
  - Transfection
  - Antibodies and Immunological Methods
  - Preparation of Matrigel-coated Polyacrylamide (PAA) Hydrogels and Glass Coverslips
  - Traction Force Microscopy
  - FRET Imaging
- QUANTIFICATION AND STATISTICAL ANALYSIS
  - Quantification of FRET and TFM Images
  - Junction Formation and Cell Area Quantification
  - Statistics and Reproducibility

SUPPLEMENTAL INFORMATION

Supplemental Information can be found online at https://doi.org/10.1016/j.celrep.2020.107924.

ACKNOWLEDGMENTS

We are very grateful for the advice of Dr. Martial Balland, his support to set up the TFM analysis, and his sharing of his TFM MATLAB script. This work was supported by the BBSRC (BB/N014855/1 and BB/N001133/1) and by the German Research Foundation to K.E. (EB160/7-1).

AUTHOR CONTRIBUTIONS

A.J.H. and K.M. performed experiments. C.H. and K.E. generated JAM-A reagents. A.R. helped set up the TFM analysis and co-designed the TFM MATLAB script. A.J.H., C.Z., M.T., M.S.B., and K.M. designed the project and drafted the manuscript. All authors read and contributed to the final version of the manuscript.

DECLARATION OF INTERESTS

The authors declare no competing interests.

Received: November 20, 2019
Revised: May 8, 2020
Accepted: June 26, 2020
Published: July 21, 2020

REFERENCES

Acharya, B.R., Nestor-Bergmann, A., Liang, X., Gupta, S., Duszyc, K., Gauquelin, E., Gomez, G.A., Budnar, S., Marcq, P., Jensen, O.E., et al. (2018). A Mechanosensitive RhoA Pathway that Protects Epithelia against Acute Tensile Stress. Dev. Cell *47*, 439–452.e436.

Aigouy, B., Umetsu, D., and Eaton, S. (2016). Segmentation and Quantitative Analysis of Epithelial Tissues. Methods Mol. Biol. *1478*, 227–239.

Aijaz, S., Sanchez-Heras, E., Balda, M.S., and Matter, K. (2007). Regulation of tight junction assembly and epithelial morphogenesis by the heat shock protein Apg-2. BMC Cell Biol. *8*, 49.

Alkasalias, T., Alexeyenko, A., Hennig, K., Danielsson, F., Lebbink, R.J., Fielden, M., Turunen, S.P., Lehti, K., Kashuba, V., Madapura, H.S., et al. (2017). RhoA knockout fibroblasts lose tumor-inhibitory capacity in vitro and promote tumor growth in vivo. Proc. Natl. Acad. Sci. USA *114*, E1413–E1421.

Angulo-Urarte, A., van der Wal, T., and Huveneers, S. (2020). Cell-cell junctions as sensors and transducers of mechanical forces. Biochim. Biophys. Acta Biomembr. *862*, 183316.

Asano, Y., and Mabuchi, I. (2001). Calyculin-A, an inhibitor for protein phosphatases, induces cortical contraction in unfertilized sea urchin eggs. Cell Motil. Cytoskeleton *48*, 245–261.

Balda, M.S., and Anderson, J.M. (1993). Two classes of tight junctions are revealed by ZO-1 isoforms. Am. J. Physiol. *264*, C918–C924.

Balda, M.S., and Matter, K. (2016). Tight junctions as regulators of tissue remodelling. Curr. Opin. Cell Biol. *42*, 94–101.

Balda, M.S., Whitney, J.A., Flores, C., González, S., Cereijido, M., and Matter, K. (1996). Functional dissociation of paracellular permeability and transepithelial electrical resistance and disruption of the apical-basolateral intramembrane diffusion barrier by expression of a mutant tight junction membrane protein. J. Cell Biol. *134*, 1031–1049.

Bartkiewicz, M., Kazazić, S., Krasowska, J., Clark, P.L., Wielgus-Kutrowska, B., and Bzowska, A. (2018). Non-fluorescent mutant of green fluorescent protein sheds light on the mechanism of chromophore formation. FEBS Lett. *592*, 1516–1523.

Benais-Pont, G., Punn, A., Flores-Maldonado, C., Eckert, J., Raposo, G., Fleming, T.P., Cereijido, M., Balda, M.S., and Matter, K. (2003). Identification of a tight junction-associated guanine nucleotide exchange factor that activates Rho and regulates paracellular permeability. J. Cell Biol. *160*, 729–740.

Beutel, O., Maraspini, R., Pombo-Garcia, K., Martin-Lemaitre, C., and Honigmann, A. (2019). Phase Separation of Zonula Occludens Proteins Drives Formation of Tight Junctions. Cell *179*, 923–936.e911.

(C–E) JAM-A and the Rho GEFs p114RhoGEF and GEF-H1 were depleted individually or combined as indicated prior to immunoblotting (C) or immunofluorescence (D). (D) Shows staining for double-phosphorylated MLC, a quantification of which is shown in (E) (n = 73 for all categories; shown are datapoints and means ± 1 SD). See Figure S3B for images of F-actin and single-phosphorylated MLC.
(F and G) Tight junction formation was assessed by quantifying occludin staining disruptions in siRNA-transfected cells plated on Matrigel-coated glass coverslips or 40 kPa or 1 kPa hydrogels (F). Examples of disrupted tight junctions are labeled by arrowheads. Numbers of junctional disruptions per 100 cells were manually counted (datapoints correspond to single fields analyzed; also shown are the median and the interquartile range; >100 cells were analyzed for each category) (G).
(H) Scheme summarizing the mechanism of regulation of tensile stress on tight junctions by JAM-A and ECM. Magnification bars, 20 μm. See also Figures S3 and S4.

CellPress
OPEN ACCESS

Borghi, N., Sorokina, M., Shcherbakova, O.G., Weis, W.I., Pruitt, B.L., Nelson, W.J., and Dunn, A.R. (2012). E-cadherin is under constitutive actomyosin-generated tension that is increased at cell-cell contacts upon externally applied stretch. Proc. Natl. Acad. Sci. USA *109*, 12568–12573.

Butler, J.P., Tolić-Nørrelykke, I.M., Fabry, B., and Fredberg, J.J. (2002). Traction fields, moments, and strain energy that cells exert on their surroundings. Am. J. Physiol. Cell Physiol. *282*, C595–C605.

Cartagena-Rivera, A.X., Van Itallie, C.M., Anderson, J.M., and Chadwick, R.S. (2017). Apical surface supracellular mechanical properties in polarized epithelium using noninvasive acoustic force spectroscopy. Nat. Commun. *8*, 1030.

Charras, G., and Yap, A.S. (2018). Tensile Forces and Mechanotransduction at Cell-Cell Junctions. Curr. Biol. *28*, R445–R457.

Ebnet, K., Schulz, C.U., Meyer Zu Brickwedde, M.K., Pendl, G.G., and Vestweber, D. (2000). Junctional adhesion molecule interacts with the PDZ domain-containing proteins AF-6 and ZO-1. J. Biol. Chem. *275*, 27979–27988.

Fanning, A.S., and Anderson, J.M. (2009). Zonula occludens-1 and -2 are cytosolic scaffolds that regulate the assembly of cellular junctions. Ann. N Y Acad. Sci. *1165*, 113–120.

Galbraith, C.G., and Sheetz, M.P. (1998). Forces on adhesive contacts affect cell function. Curr. Opin. Cell Biol. *10*, 566–571.

Grashoff, C., Hoffman, B.D., Brenner, M.D., Zhou, R., Parsons, M., Yang, M.T., McLean, M.A., Sligar, S.G., Chen, C.S., Ha, T., and Schwartz, M.A. (2010). Measuring mechanical tension across vinculin reveals regulation of focal adhesion dynamics. Nature *466*, 263–266.

Hatte, G., Prigent, C., and Tassan, J.P. (2018). Tight junctions negatively regulate mechanical forces applied to adherens junctions in vertebrate epithelial tissue. J. Cell Sci. *131*, jcs208736.

Henson, J.H., Kolnik, S.E., Fried, C.A., Nazarian, R., McGreevy, J., Schulberg, K.L., Detweiler, M., and Trabosh, V.A. (2003). Actin-based centripetal flow: phosphatase inhibition by calyculin-A alters flow pattern, actin organization, and actomyosin distribution. Cell Motil. Cytoskeleton *56*, 252–266.

Kreis, T.E. (1987). Microtubules containing detyrosinated tubulin are less dynamic. EMBO J. *6*, 2597–2606.

Lye, M.F., Fanning, A.S., Su, Y., Anderson, J.M., and Lavie, A. (2010). Insights into regulated ligand binding sites from the structure of ZO-1 Src homology 3-guanylate kinase module. J. Biol. Chem. *285*, 13907–13917.

Matter, K., Whitney, J.A., Yamamoto, E.M., and Mellman, I. (1993). Common signals control low density lipoprotein receptor sorting in endosomes and the Golgi complex of MDCK cells. Cell *74*, 1053–1064.

Milloud, R., Destaing, O., de Mets, R., Bourrin-Reynard, I., Oddou, C., Delon, A., Wang, I., Albigès-Rizo, C., and Balland, M. (2017). $\alpha v\beta 3$ integrins negatively regulate cellular forces by phosphorylation of its distal NPXY site. Biol. Cell *109*, 127–137.

Monteiro, A.C., Sumagin, R., Rankin, C.R., Leoni, G., Mina, M.J., Reiter, D.M., Stehle, T., Dermody, T.S., Schaefer, S.A., Hall, R.A., et al. (2013). JAM-A associates with ZO-2, afadin, and PDZ-GEF1 to activate Rap2c and regulate epithelial barrier function. Mol. Biol. Cell *24*, 2849–2860.

Nie, M., Aijaz, S., Leefa Chong San, I.V., Balda, M.S., and Matter, K. (2009). The Y-box factor ZONAB/DbpA associates with GEF-H1/Lfc and mediates Rho-stimulated transcription. EMBO Rep. *10*, 1125–1131.

Niessen, C.M., and Gottardi, C.J. (2008). Molecular components of the adherens junction. Biochim. Biophys. Acta *1778*, 562–571.

Otani, T., Nguyen, T.P., Tokuda, S., Sugihara, K., Sugawara, T., Furuse, K., Miura, T., Ebnet, K., and Furuse, M. (2019). Claudins and JAM-A coordinately regulate tight junction formation and epithelial polarity. J. Cell Biol. *218*, 3372–3396.

Peddibhotla, S.S., Brinkmann, B.F., Kummer, D., Tuncay, H., Nakayama, M., Adams, R.H., Gerke, V., and Ebnet, K. (2013). Tetraspanin CD9 links junctional adhesion molecule-A to $\alpha v\beta 3$ integrin to mediate basic fibroblast growth factor-specific angiogenic signaling. Mol. Biol. Cell *24*, 933–944.

Schwayer, C., Shamipour, S., Pranjic-Ferscha, K., Schauer, A., Balda, M., Tada, M., Matter, K., and Heisenberg, C.P. (2019). Mechanosensation of Tight Junctions Depends on ZO-1 Phase Separation and Flow. Cell *179*, 937–952.e918.

Scott, D.W., Tolbert, C.E., and Burridge, K. (2016). Tension on JAM-A activates RhoA via GEF-H1 and p115 RhoGEF. Mol. Biol. Cell *27*, 1420–1430.

Spadaro, D., Le, S., Laroche, T., Mean, I., Jond, L., Yan, J., and Citi, S. (2017). Tension-Dependent Stretching Activates ZO-1 to Control the Junctional Localization of Its Interactors. Curr. Biol. *27*, 3783–3795.e3788.

Steed, E., Elbediwy, A., Vacca, B., Dupasquier, S., Hemkemeyer, S.A., Suddason, T., Costa, A.C., Beaudry, J.B., Zihni, C., Gallagher, E., et al. (2014). MarvelD3 couples tight junctions to the MEKK1-JNK pathway to regulate cell behavior and survival. J. Cell Biol. *204*, 821–838.

Steger, C. (1998). An unbiased detector of curvilinear structures. IEEE Trans. Pattern Anal. Mach. Intell. *20*, 113–125.

Tang, X., Ali, M.Y., and Saif, M.T. (2012). A Novel Technique for Micro-patterning Proteins and Cells on Polyacrylamide Gels. Soft Matter *8*, 7197–7206.

Terry, S.J., Zihni, C., Elbediwy, A., Vitiello, E., Leefa Chong San, I.V., Balda, M.S., and Matter, K. (2011). Spatially restricted activation of RhoA signalling at epithelial junctions by p114RhoGEF drives junction formation and morphogenesis. Nat. Cell Biol. *13*, 159–166.

Tornavaca, O., Chia, M., Dufton, N., Almagro, L.O., Conway, D.E., Randi, A.M., Schwartz, M.A., Matter, K., and Balda, M.S. (2015). ZO-1 controls endothelial adherens junctions, cell-cell tension, angiogenesis, and barrier formation. J. Cell Biol. *208*, 821–838.

Tsapara, A., Matter, K., and Balda, M.S. (2006). The heat-shock protein Apg-2 binds to the tight junction protein ZO-1 and regulates transcriptional activity of ZONAB. Mol. Biol. Cell *17*, 1322–1330.

Tse, J.R., and Engler, A.J. (2010). Preparation of hydrogel substrates with tunable mechanical properties. Curr. Protoc. Cell Biol. *Chapter 10*, Unit 10.16.

Tuncay, H., Brinkmann, B.F., Steinbacher, T., Schürmann, A., Gerke, V., Iden, S., and Ebnet, K. (2015). JAM-A regulates cortical dynein localization through Cdc42 to control planar spindle orientation during mitosis. Nat. Commun. *6*, 8128.

Van Itallie, C.M., and Anderson, J.M. (1997). Occludin confers adhesiveness when expressed in fibroblasts. J. Cell Sci. *110*, 1113–1121.

Varadarajan, S., Stephenson, R.E., and Miller, A.L. (2019). Multiscale dynamics of tight junction remodeling. J. Cell Sci. *132*, jcs229286.

Vitiello, E., Moreau, P., Nunes, V., Mettouchi, A., Maiato, H., Ferreira, J.G., Wang, I., and Balland, M. (2019). Acto-myosin force organization modulates centriole separation and PLK4 recruitment to ensure centriole fidelity. Nat. Commun. *10*, 52.

Yoshizaki, H., Ohba, Y., Kurokawa, K., Itoh, R.E., Nakamura, T., Mochizuki, N., Nagashima, K., and Matsuda, M. (2003). Activity of Rho-family GTPases during cell division as visualized with FRET-based probes. J. Cell Biol. *162*, 223–232.

Zihni, C., Munro, P.M., Elbediwy, A., Keep, N.H., Terry, S.J., Harris, J., Balda, M.S., and Matter, K. (2014). Dbl3 drives Cdc42 signaling at the apical margin to regulate junction position and apical differentiation. J. Cell Biol. *204*, 111–127.

Zihni, C., Mills, C., Matter, K., and Balda, M.S. (2016). Tight junctions: from simple barriers to multifunctional molecular gates. Nat. Rev. Mol. Cell Biol. *17*, 564–580.

Zihni, C., Vlassaks, E., Terry, S., Carlton, J., Leung, T.K.C., Olson, M., Pichaud, F., Balda, M.S., and Matter, K. (2017). An apical MRCK-driven morphogenetic pathway controls epithelial polarity. Nat. Cell Biol. *19*, 1049–1060.

STAR★METHODS

KEY RESOURCES TABLE

REAGENT or RESOURCE	SOURCE	IDENTIFIER
Antibodies		
Mouse monoclonal anti-Occludin	ThermoFisher Scientific	Cat#331500; RRID:AB_2533101
Mouse monoclonal anti-ZO-1	ThermoFisher Scientific	Cat#339100; RRID:AB_87181
Mouse monoclonal anti-p-MLC S19	Cell Signaling Technology	Cat#3675; RRID:AB_2250969
pp-MLC Thr18S19, rabbit polyclonal	Cell Signaling Technology	Cat#3674; RRID:AB_2147464
Rabbit polyclonal anti-p114RhoGEF	abcam	Cat#ab96520; RRID:AB_10680897
Rabbit polyclonal anti-p114RhoGEF	GeneTex	Cat#GTX102223; RRID:AB_1949683
Sheep polyclonal anti-β-catenin	abcam	Cat#ab65747; RRID:AB_1140675
Goat polyclonal anti-p120catenin	Santa Cruz Biotechnology	Cat#sc-373116
Mouse monoclonal anti-Flag M2	Sigma-Aldrich	Cat#F-3165; RRID:AB_259529
Rabbit polyclonal anti-myosin-IIA	Sigma-Aldrich	Cat#M8064; RRID:AB_260673
Mouse monoclonal anti-Talin	Sigma-Aldrich	Cat#T3287; RRID:AB_477572
Mouse monoclonal anti-E-cadherin	BD Biosciences	Cat#610182; RRID:AB_397581
Mouse monoclonal anti-GFP	Abgent	Cat#AM1009a; RRID:AB_352468
Rabbit polyclonal anti-ZO-1	Benais-Pont et al., 2003	N/A
Rabbit polyclonal anti-JAM-A	Tuncay et al., 2015	N/A
Mouse monoclonal anti-GEF-H1	Benais-Pont et al., 2003	N/A
Rabbit polyclonal anti-GEF-H1	Benais-Pont et al., 2003	N/A
Mouse monoclonal anti-α-tubulin	Kreis, 1987	N/A
Alexa488-Donkey anti-mouse IgG	Jackson ImmunoResearch	Cat#715-545-150; RRID:AB_2340846
Cy3-Donkey anti-rabbit IgG	Jackson ImmunoResearch	Cat#711-165-152; RRID:AB_2307443
Alexa64-Donkey anti-goat IgG	Jackson ImmunoResearch	Cat#705-605-147; RRID:AB_2340437
Cy3-Donkey anti-mouse IgG	Jackson ImmunoResearch	Cat#715-165-150; RRID:AB_2340813
FITC-Donkey anti-sheep IgG	Jackson ImmunoResearch	Cat#713-095-147; RRID:AB_2340719
HRP-Goat anti-rabbit IgG	Jackson ImmunoResearch	Cat#111-035-003; RRID:AB_2313567
HRP-Goat anti-mouse IgG	Jackson ImmunoResearch	Cat#115-005-003; RRID:AB_2338447
IRDye 800CW-Donkey anti-mouse IgG	LI-COR	Cat#926-32212; RRID:AB_621847
IRDye 680LT-Donkey anti-rabbit IgG	LI-COR	Cat#926-68023; RRID:AB_10706167
Chemicals, Peptides, and Recombinant Proteins		
Phalloidin-Atto647	Sigma-Aldrich	Cat#65906
Blebbistatin	Tocris Bioscience	Cat#1760
Y27632	Tocris Bioscience	Cat#1254
RNAiMAX	ThermoFisher Scientific	Cat#13778150
TransIT	Mirus Bio	Cat#MIR6000
Prolong Gold antifade reagent	ThermoFisher Scientific	Cat#P36930
Carboxyl polystyrene beads, 0.20 μm, Dragon Green	Bang Laboratories	Cat#FCDG003
Experimental Models: Cell Lines		
Dog: MDCK	Matter et al., 1993	N/A
Dog: MDCK h-p114RhoGEF	Terry et al., 2011	N/A
Dog: MDCK h-p114RhoGEF Y-A260	Terry et al., 2011	N/A
Recombinant DNA		
Tension sensor (TS) module	Addgene	Cat#26021; RRID:Addgene_26021
pcDNA-TO-B	ThermoFisher Scientific	Cat#V385-20
pcDNA-TO-TS	This paper	N/A

(*Continued on next page*)

Continued

REAGENT or RESOURCE	SOURCE	IDENTIFIER
pcDNA-TO-ZO-1-TS	This paper	N/A
pcDNA-TO-ZO-1-TSΔCTD	This paper	N/A
pcDNA-TO-ZO-1-TSIΔ	This paper	N/A
pcDNA-TO-ZO-1-ΔVFP	This paper	N/A
pRaichu-RhoA	Yoshizaki et al., 2003	N/A
pTS-E-cadherin	Borghi et al., 2012	N/A
pTS-E-cadherinΔCTD	Borghi et al., 2012	N/A
pFlag-CMV1-JAM-A	Peddibhotla et al., 2013	N/A

RESOURCE AVAILABILITY

Lead Contact

Further information and requests for resources, reagents, and data should be addressed to the Lead Contact, Karl Matter (k.matter@ucl.ac.uk).

Materials Availability

Plasmids generated in this study will be made available on request from the Lead Contact, but we may require a shipping payment and completed Materials Transfer Agreement.

Data and Code Availability

The datasets supporting the current study have not been deposited in a public repository because all data collected are included in the study. Data are available from the Lead Contact upon reasonable request. No new code was developed for this study.

EXPERIMENTAL MODEL AND SUBJECT DETAILS

MDCK cells were grown in DMEM supplemented with 10% fetal bovine serum (Matter et al., 1993). MDCK lines expressing h-p114RhoGEF or h-p114RhoGEF Y-A260 were described previously (Terry et al., 2011). Fresh batches of cells from a contamination-free stock that had been tested for mycoplasma were used to replace fresh cultures every 6 to 8 weeks. Cells were then weekly stained with Hoechst dye to reveal nuclei and DNA of contaminants such as mycoplasma.

METHOD DETAILS

Small Molecule Inhibitors

Blebbistatin and the ROCKI/II inhibitor Y27632 were used at 10μM final concentration diluted from 10mM stocks in DMSO, and Calyculin A at 20nM diluted from a 20μM stock in DMSO.

Expression Plasmids

The tension sensor (TS) module was a gift from Martin Schwartz (http://n2t.net/addgene:26021; RRID:Addgene_26021) (Grashoff et al., 2010). The tension sensor module was amplified by PCR and cloned into the pcDNA-TO-B plasmid using the EcoRI and NotI site either without or with a stop codon at the 3′ end (primers: 5′-CCAAAATGTCGTAACAACTCCGCCCCATTGACG-3′, 5′-GCTCGA GCGGCCGCTTACTTGTACAGCTCGTCCATGCCGAGAGTGATCC-3′ and 5′-GCTCGAGCGGCC GCCTTGTACAGCTCGT CCATGCCGAGAGTGATCC-3′), resulting in pcDNA-TO-TS. To construct ZO-1-TS, the human alpha+ cDNA was used as a template to generate a fragment containing the N-terminal half until residue 806 with BamH1 and EcoRI sites for cloning (5′-ATTCGCG GATCCA TGGAGGAAACAGCTATATGGGAAC-3′ and 5′-ATTCGCGAATTCATCATCATGCAAATCAAGGTC ATC-3′), and the fragment was cloned into pcDNA-TO-TS with and without the STOP codon (Balda and Anderson, 1993). The CTD was amplified accordingly using 5′-GACACGGATGCG GCCGCTCGTCTGTCCTACCTGTCAGCTCCAGG-3′ and 5′-GAGATTCCACCGGTTAAAAGTGG TCAATAAGGACAGAAACAC-3′ and cloned into pcDNA-TO-TS without stop containing the N-terminal half of ZO-1 using the NotI site and a blunted SacII site. To inactivate intramolecular FRET, non-fluorescent mTFP was generated by deleting residues 70-75, which are within the region required for fluorescence (Bartkiewicz et al., 2018), by generating two overlapping PCR fragments with 5′-GACCACCGCGGCCTTCACCAAGTAC-3′ and 5′-GACAGGTAGGACAGACGAG CGGCCGC-3′; and, 5′-TGG TGAAGGCCGCGGTGGTCAGAATG-3′ and 5′- GAACAGAGC TGAGCAGCTAGCCAGT-3′ using pcDNA-ZO-1-TS as a template. A construct lacking VFP was synthesized by generating a fragment with 5′- GAACAGAGCTGAGCAGCTAGCCAGT −3′ and 5′-GG TAGGACAGACGAGCGGCCGCCCCTGCACCACCTGGCCCCTTGTA-3′. The purified PCR products were then cloned into a NheI

site within the ZO-1 cDNA and the Not1 site at the junction between the FRET module and the CTD of ZO-1 using the In-Fusion cloning method (Takara Bio). pRaichu-RhoA was kindly provided by Michiyuki Matsuda (Osaka University, Japan) (Yoshizaki et al., 2003) and canine E-cadherin tension sensor constructs by Alexander Dunn (Stanford University, USA) (Borghi et al., 2012). The Flag-tagged human JAM-A cDNA cloned into pFlag-CMV1 has been described previously (Peddibhotla et al., 2013).

Transfection

Cells were cultured and transfected with siRNAs using RNAiMAX transfection reagent following the manufacturer's instructions using 1μl per 48-plate well and 1μl of a 20μM siRNA stock. Transfections were left for 24 hours before the mixture was replaced with fresh medium. For experiments on coated coverslips, 2.5×10^5 cells/well were plated into 6-well plates and transfected with 8μl/well RNAiMAX and 8μl/well of 20μM siRNA. The following day, the cells were plated onto the extracellular matrices required for the experiment. The following siRNAs were used: canine JAM-A 5′-CCAGUAAGAAGGUGAUUUA-3′ and 5′-CAUCCAAGCC CACGGUCAA-3′; canine ARHGEF18/ p114RhoGEF 5′-AAGACCACGUCGGGACGCUUG-3′ and 5′-AACUACGUCAUCCAGAAAAUC-3′; and canine ARHGEF2/GEF-H1 5′-AGACACAGGACGAGGCUUA-3′, 5′-GGGAAAAGGAGAAGAU GAA-3′ and 5′-GUGCGGAGCGGAUGCGCG UAA-3′. DNA was transfected using TransIT using 1μl per 48-plate well and 1μg of DNA. Transfections were left for 4 hours before the mixture was replaced with fresh medium.

Antibodies and Immunological Methods

Fixation and processing of cells for immunofluorescence and immunoblotting were as previously described (Balda et al., 1996; Zihni et al., 2014, 2017). The following commercial antibodies were used: occludin, mouse monoclonal, and ZO-1 mouse monoclonal, 1/1000 for immunofluorescence; p-MLC S19, mouse monoclonal and pp-MLC Thr18, S19 rabbit polyclonal, immunofluorescence 1/100 and 1/200, respectively, and immunoblotting 1/1000; p114RhoGEF, rabbit polyclonal, immunofluorescence 1/300 and immunoblotting 1/1000; anti-β-catenin, sheep polyclonal, immunofluorescence 1/400; p120catenin, goat polyclonal polyclonal, immunofluorescence 1/300; Flag M2, mouse monoclonal, immunofluorescence 1/4000; nonmuscle myosin-IIA, rabbit polyclonal, immunofluorescence 1/1000; talin, mouse monoclonal, immunofluorescence 1/1000; E-cadherin, mouse monoclonal, immunofluorescence 1/500; GFP, mouse monoclonal, immunoblotting 1/300. Affinity-purified rabbit polyclonal antibodies were used for ZO-1, immunofluorescence (1/1000) (Benais-Pont et al., 2003) and JAM-A (1/1000) (Tuncay et al., 2015). Mouse monoclonal (1/20) and rabbit polyclonal (1/1000) anti-GEF-H1 antibodies were described previously (Benais-Pont et al., 2003). Mouse monoclonal anti-α-tubulin 1A2 was used for immunblotting (1/20) (Kreis, 1987). Phalloidin-Atto647 was from Sigma-Aldrich and diluted 1/1000. Affinity-purified and cross-adsorbed Alexa488-, Cy3- and Cy5-labeled donkey anti-mouse, rabbit, and goat secondary antibodies were diluted 1/300 from 50% glycerol stocks. Affinity-purified HRP-conjugated goat anti mouse and rabbit were diluted 1/5000 from 50% glycerol stocks. For immunofluorescence analysis, cells were mounted using Prolong Gold antifade reagent. Imaging was performed using a Nikon Eclipse Ti-E microscope with a CFI Apochromat Nano-Crystal 60x oil lens (N.A., 1.2) or a Leica TCS SP8 with an HC PL APO 40x oil lens (N.A., 1.30). Images were processed using ImageJ/Fiji and Adobe Photoshop CC software. Quantifications of junctional recruitment were performed with ImageJ/Fiji using the plot profile tool to measure fluorescence intensity peaks of two opposing junctions per cell, which were then averaged for each cell and normalized to the mean integrated density of the entire image. Immunoblotting was carried out using methods previously described (Steed et al., 2014; Zihni et al., 2014). Immunoblots were quantified with ImageJ/Fiji.

Preparation of Matrigel-coated Polyacrylamide (PAA) Hydrogels and Glass Coverslips

The PAA hydrogel fabrication method was adapted from a previously described protocol (Vitiello et al., 2019). 22, 13 and 10mm glass coverslips (Agar Scientific) were rinsed with 70% ethanol, dried at 70°C, and then heated with a Bunsen burner set on a blue flame to sterilize the coverslips and render the surface hydrophilic. 22 and 10mm coverslips were then incubated with a solution of 65 μg/ml Matrigel (BD Biosciences) for 2.5 hours at 37°C. 13mm coverslips were silanized for 3 minutes using ethanol containing 0.37% Bind-Silane solution (GE Healthcare Life Science) and 3.2% acetic acid. PAA and bis-acrylamide (N,N′-methylenebisacrylamide, Sigma-Aldrich) mixes corresponding to E-moduli of 1 kPa and 40 kPa were prepared following established guidelines (Tse and Engler, 2010). For TFM, PAA hydrogels with an E-modulus ranging from 13.6 to 19.6 kPa (average of 16.4 ± 2.5 kPa) were fabricated by adding 1 μL fluorescent carboxyl polystyrene beads (diameter = 0.20 μm; Dragon Green) to the PAA/bis-AA mix. Mixes were sonicated and vortexed to avoid bead clumps and to ensure a homogeneous distribution of the beads in the gels. N,N,N′,N′-tetramethylethylenediamine (TEMED, Sigma) and 10% APS (Sigma) were added to PAA/bis-PAA mixes at 0.1% and 0.8%, respectively, to prime polymerization, and a 25 μL drop of activated mix was added on each coated 22 mm coverslips. Silanized 13mm coverslips were then quickly put on top of the drops, and gels were let to polymerize for 30 minutes, allowing the transfer of matrix protein from the coated glass coverslip to the surface of the polymerizing gel, following the principle behind microcontact printing on PAA gels (μCP) (Tang et al., 2012). After polymerization, the assemblies were soaked in sterile H_2O for 10 minutes. The gels attached to the top coverslips were then detached from the bottom coverslips using a scalpel blade. Gels bound to coverslips were stored at 4°C in sterile H_2O containing 150mM NaCl and 10mM HEPES and incubated in culture medium for 30 min at 37°C prior to cell seeding.

Traction Force Microscopy

Static TFM imaging was performed on a Nikon Eclipse Ti-E microscope using a Super Plan Fluor ELWD 20x objective (N.A. 0.45) using the Nikon Perfect Focus system. To obtain well separated islands of 10 to 20 cells that would allow acquisition of images

with a sufficient margin of surrounding matrix without cells, which is required for the analysis (no-displacement fields), 1 x10^3 to 3 x10^3 cells transfected with siRNAs were seeded on hydrogels two days before imaging. Briefly, islands were imaged in bright field and image stacks of the beads embedded in the contracted substrate were taken in the FITC channel. Cell were then lysed with 1M NaOH + 1% sodium dodecyl sulfate and image stacks of the beads in the relaxed substrate were taken.

FRET Imaging

For RhoA activity assays, cells were plated into ibid 8 multi-well chamber slides and then transfected with pRaichu-RhoA. For tension sensor experiments, 7 x10^3 cells were seeded on Matrigel-coated 10 mm glass coverslips, 12.5 x10^3 cells on 40 kPa hydrogels and 25 x10^3 cells on 1 kPa hydrogels. The RhoA probe and the tension sensors were then transfected and the assays were performed 20 to 24 hours later. The FRET analysis was performed at 37°C with a Nikon Eclipse Ti-E inverted microscope equipped with excitation and emission CFP and YFP filters in external filter wheels and using a CFI Apochromat Nano-Crystal 60x oil lens (N.A., 1.2). Crossover between CFP and YFP filters was calibrated by imaging individual fluorescent proteins expressed alone using all four emission/excitation filter combinations. FRET efficiency maps were then generated with the Nikon software using the built-in formula to correct for crossover between CFP and YFP channels. For donor recovery after acceptor bleaching experiments, a Leica SP2 microscope (63x objective, 37°C, N.A., 1.4) and Leica FRET software using the donor recovery after acceptor bleaching (YFP was bleached to 40%) protocol (Terry et al., 2011). FRET efficiency maps were then generated using the Leica software by calculating the FRET efficiency according to the formula $[(D_{post}-D_{pre})/D_{post}]*100$ (D represents donor intensity; values were corrected for the bleaching level).

QUANTIFICATION AND STATISTICAL ANALYSIS

Quantification of FRET and TFM Images

For quantification of FRET images, ImageJ/Fiji, was used to calculate mean integrated densities of junctional segments above measured backgrounds. For traction force microscopy, experimental drift was corrected using the ImageJ Template Matching plugin from Dr. Qingzong Tseng (https://sites.google.com/site/qingzongtseng/template-matching-ij-plugin). Particle image velocimetry, beads tracking, displacement field calculations (linear interpolation on a regular grid with 2.6 μm spacing), unconstrained Fourier Transform Traction Cytometry, and computation of traction maps and strain energies was performed using a MATLAB script developed by Dr. Martial Balland (Alkasalias et al., 2017; Milloud et al., 2017). Islands with an out of equilibrium force > 10% of the sum of the individual force amplitudes were discarded, and a final filtering based on island size was performed to obtain a comparable pool of island sizes between the two different analyzed conditions. Strain energy densities were obtained by dividing the total strain energy U (as defined in Butler et al. 2002) calculated from the traction and displacement fields, by the area of the islands. Absolute values of single islands were then averaged across individual gels, and expressed as fold of control.

Junction Formation and Cell Area Quantification

PFA fixed glass coverslips and PAA hydrogels (40 and 1 kPa) were stained for ZO-1, occludin, p120-catenin, and nuclei, and imaged as described above. Manual quantification of cell-cell junction disruption was done on XY maximum intensity projections of the occludin staining from acquired z stacks, using ImageJ/Fiji. A junction was counted as disrupted when the occludin staining was clearly absent between two adjacent cells. For each field, the number of cells were counted (Nucleus Counter plugin, https://imagej.net/Nucleus_Counter) followed by manual correction. Total disruptions count per field was normalized to 100 cells; each image field analyzed represents a datapoint. Cell area quantification was done using a workflow in ImageJ/Fiji, using homemade macros and published scripts. The aim of this workflow was to automate an accurate tight junction staining-based cell segmentation and allow subsequent cell morphometric analysis with minimum manual correction. Briefly, a binary mask of cell-cell junctions was obtained using the Ridge Detection plugin on maximum projection of the ZO-1 staining from acquired z stacks (Steger, 1998). The binary mask was cleaned from small cytosolic objects using the Analyze Particle tool. 4-channel RGB merged images with the best focus were then overlaid on the mask for quality control and manual correction. The Tissue Analyzer plugin was then used on the mask to obtain a single-step cell segmentation (Aigouy et al., 2016). ROIs corresponding to each cell were exported back to ImageJ/Fiji's ROI manager. ROIs at the border of the image were deleted, and cell areas were measured. The plugin also produced random color maps of the segmentations that were used in Figure 2 for improved visualization of single cells in the monolayer.

Statistics and Reproducibility

For the quantifications shown, the provided n values refer to the numbers of cells, image fields or cell islands analyzed per type of sample (this information is provided in figure legends) and are derived from either two (Figures 1, 2F, 3B, 4, S1, and S4) or three (Figures 2B and 3D; Figure S3) independent experiments. Statistical significance was tested using nonparametric Kruskal-Wallis and Wilcoxon tests to compare distributions, and Wilcoxon signed-rank tests when values were normalized and compared to a standard. The FRET experiments are shown as standard boxplots (25th to 75th percentiles, with a line indicating the median). All other quantifications show all data points along with the mean and standard deviation for each category or, where indicated, the median and interquartile ranges. Graphs and statistical calculations were generated with JMP Pro (V14).

Cell Reports, Volume *32*

Supplemental Information

Interplay between Extracellular Matrix Stiffness and JAM-A Regulates Mechanical Load on ZO-1 and Tight Junction Assembly

Alexis J. Haas, Ceniz Zihni, Artur Ruppel, Christian Hartmann, Klaus Ebnet, Masazumi Tada, Maria S. Balda, and Karl Matter

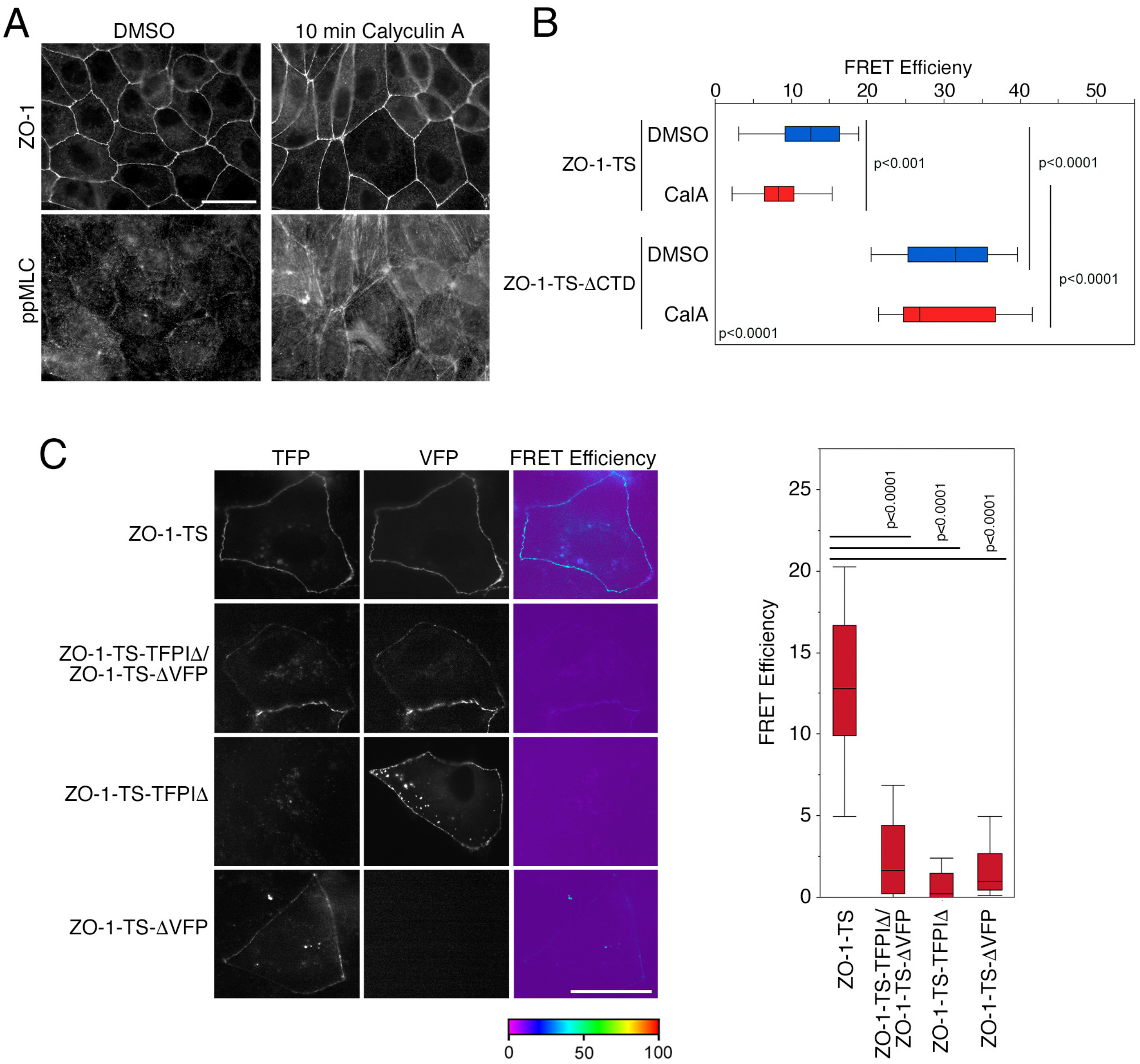

Figure S1: The ZO-1 sensor responds to increased cytoskeletal tension. Related to Figure 1.
(A) MDCK cells were treated with 20nM Calyculin A for 10 minutes prior to fixation and labelling for ZO-1 and double phosphorylated MLC. (B) FRET assay performed with cells expressing either ZO-1-TS or ZO-1-TS-ΔCTD after addition of vehicle or Calyculin A (images were taken between 10 and 20 minutes after addition; n for ZO-1-TS with DMSO, 34; ZO-1-TS-ΔCTD with DMSO, 17; ZO-1-TS with Calyculin A, 32; ZO-1-TS-ΔCTD with Calyculin A, 17; box-plot in panel E shows median and the interquartile ranges). (C) FRET assay performed with MDCK cells transfected with either ZO-1-TS, a ZO-1-TS variant with a deletion in the FRET donor TFP that renders it nonfluorescent (ZO-1-TS-TFPIΔ), a variant in which VFP had been deleted (ZO-1-TS-ΔVFP), or co-transfected with the two deletion variants (n for ZO-1-TS, 21; ZO-1-TS-TFPIΔ, 10; ZO-1-TS-ΔVFP, 21; ZO-1-TS-TFPIΔ/ZO-1-TS-ΔVFP, 20). Magnification bars, 20µm.

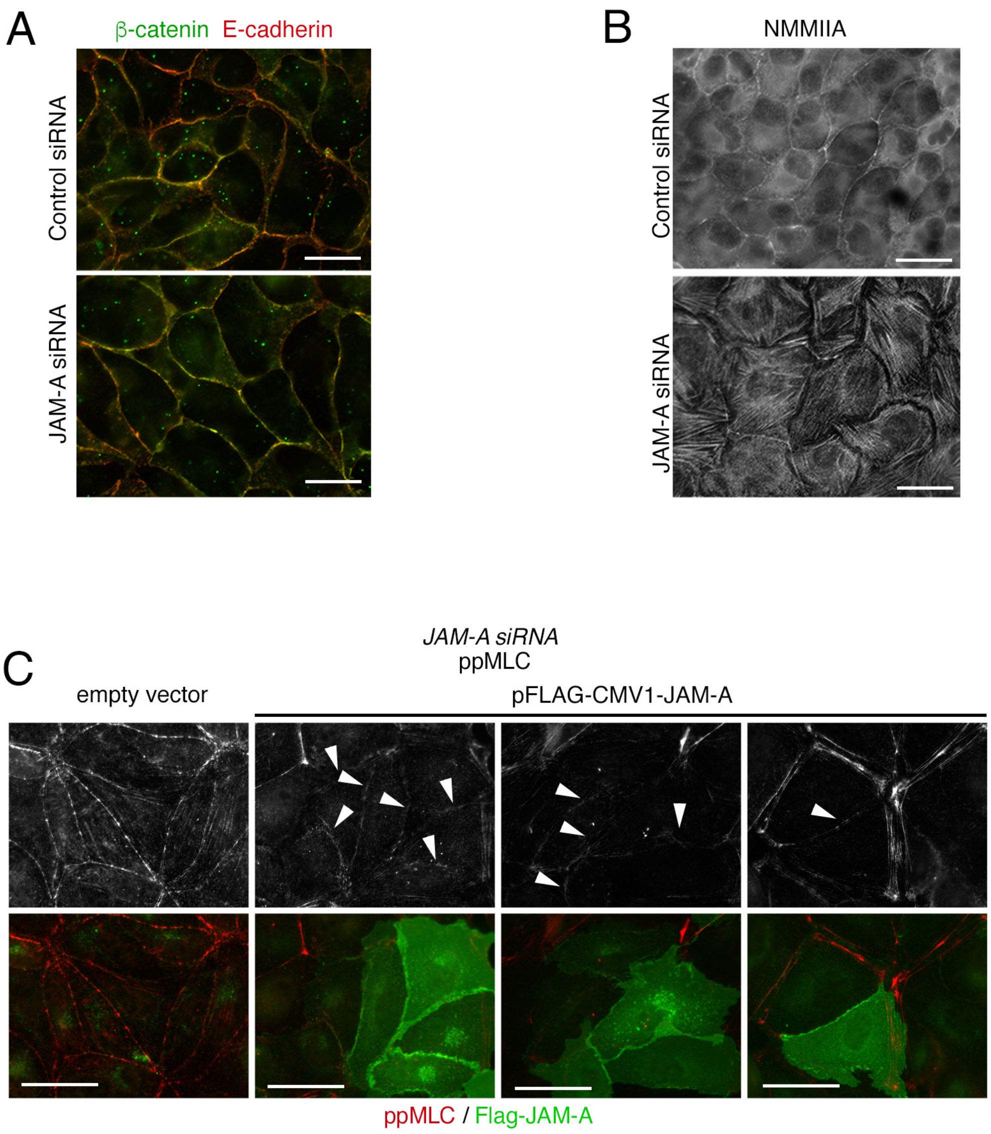

Figure S2: Effects and specificity of JAM-A depletion on MDCK cells. Related to Figure 2.
(A,B) MDCK cells were transfected with either control or JAM-A targeting siRNAs and were then fixed and stained for the adherens junction proteins E-cadherin and β-catenin (A) or the heavy chain of NMMIIA (B). (C) MDCK cells that had been transfected with JAM-A siRNAs were re-transfected with a plasmid encoding Flag-tagged human JAM-A. The cells were then stained for Flag-tagged JAM-A and double phosphorylated MLC. The first pair of images shows a field of cells transfected with an empty vector that are Flag-negative. Arrowheads label junction between cells that both re-express JAM-A and exhibit only low levels of ppMLC staining in contrast to junctions formed by cells that are JAM-A negative. Magnification bars, 20μm.

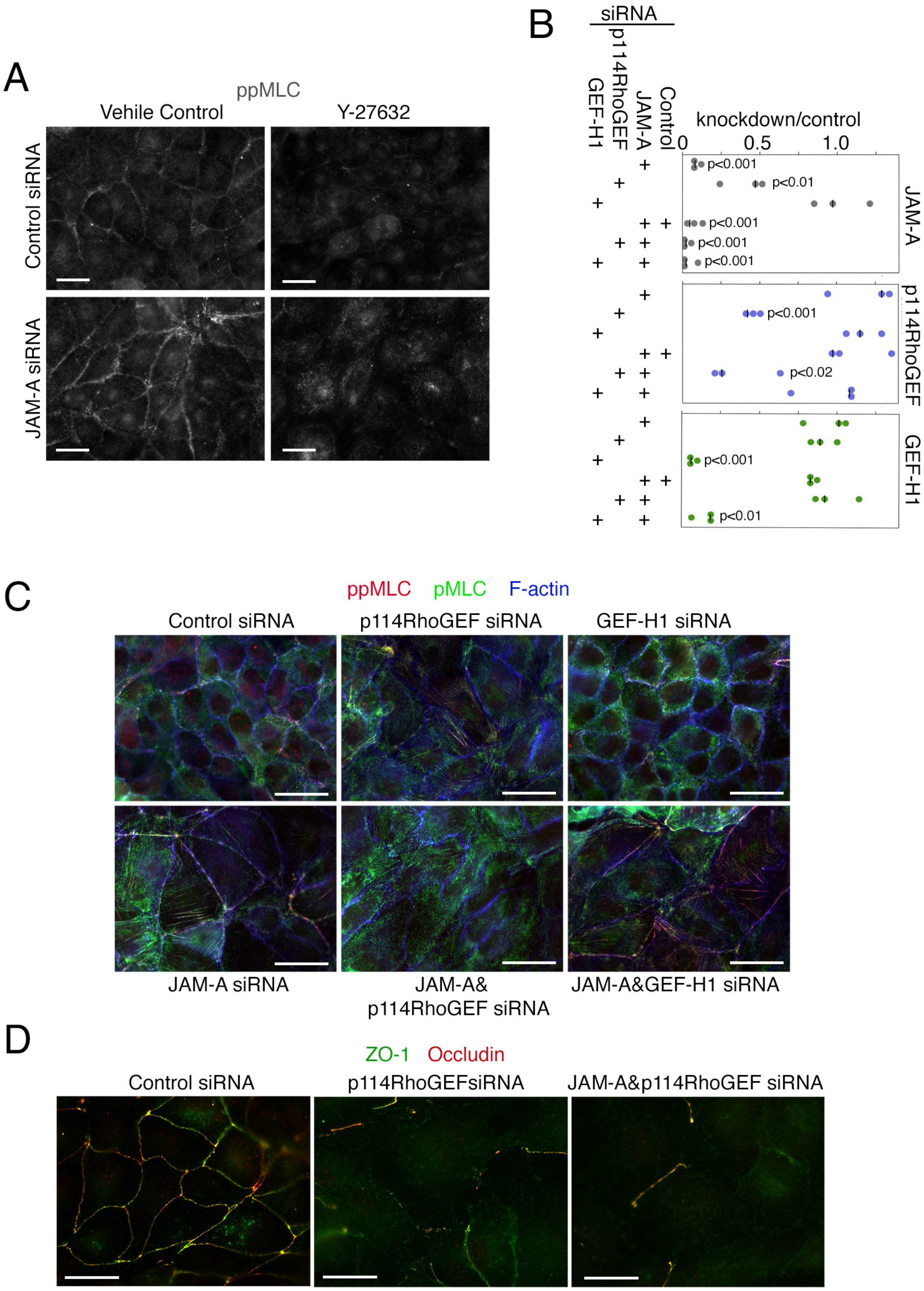

Figure S3: Analysis of signaling mechanisms downstream of JAM-A and importance of p114RhoGEF for tight junction integrity. Related to Figure 4.
(A) Control and JAM-A siRNA transfected cells were incubated with DMSO as a vehicle control or with the ROCKI/II inhibitor Y-27632 (10μM) for 5 hours prior to fixation and staining for ppMLC. (B) Quantification of immunoblots detecting JAM-A, p114RhoGEF and GEF-H1 expression. (C) Shown is an overlay of the ppMLC staining of Figure 4D with pMLC and F-actin of siRNA transfected MDCK cells. (D) MDCK cells transfected with control siRNA, p114RhoGEF siRNA, or JAM-A and p114RhoGEF siRNAs were fixed and processed for immunofluorescence using occludin and ZO-1 antibodies. Note, tight junction formation is p114RhoGEF-dependent in the presence or absence of JAM-A (cells only depleted of JAM-A are shown in Fig. 2C). Magnification bars, 20μm.

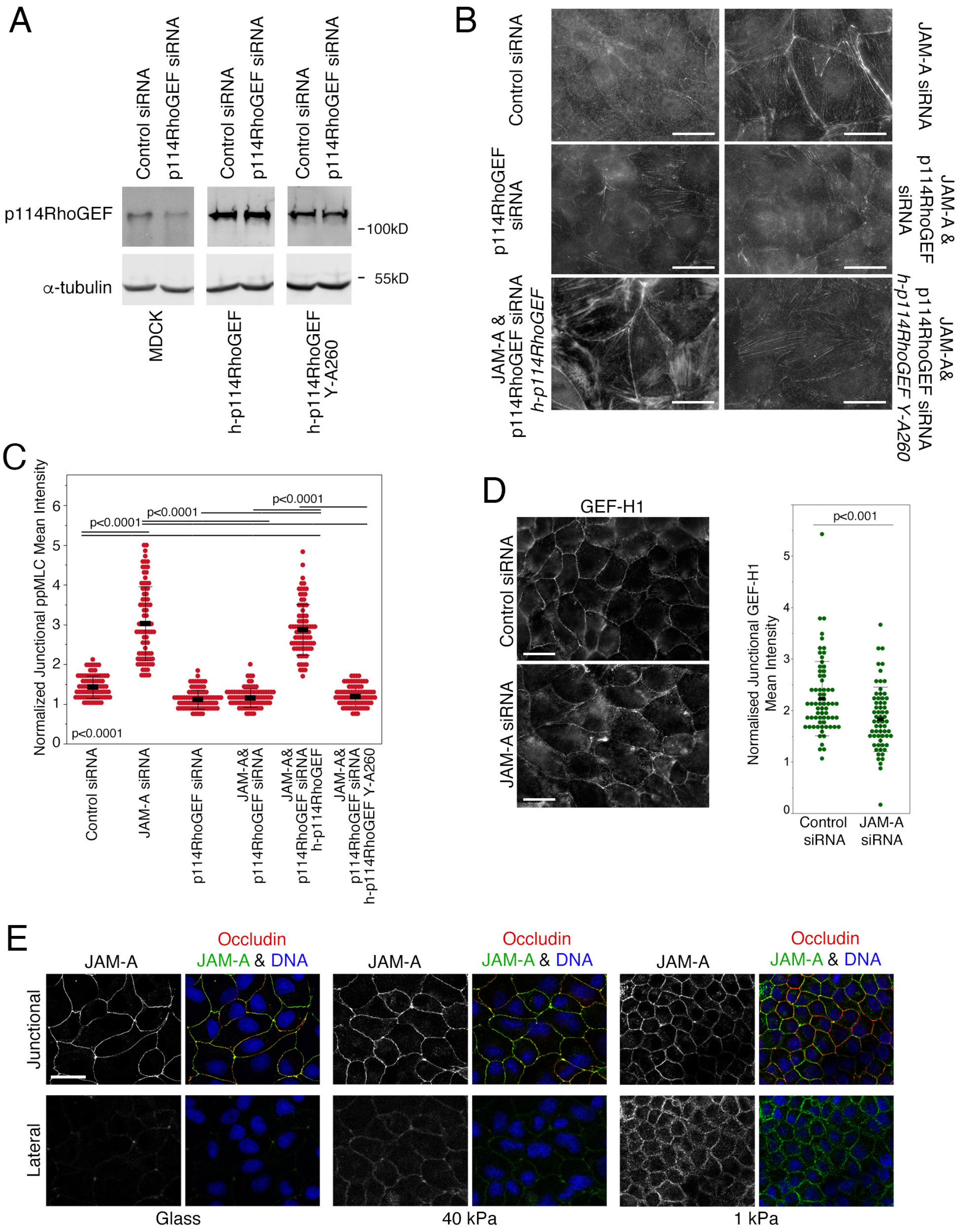

Figure S4: The catalytic activity of p114RhoGEF is required for junctional actomyosin activation in JAM-A depleted cells. Related to Figure 4.

(A-C) Rescue of junctional double phosphorylation of MLC in single or double siRNA-transfected MDCK cells was assayed after transfecting either active or inactive (Y-A260) human p114RhoGEF. Panel A shows an immunoblot demonstrating that human p114RhoGEF is not affected by the siRNAs that reduce expression of canine p114RhoGEF. Panel B shows immunofluorescence images of cells stained for double phosphorylated MLC. Panel C shows quantification of images from the experiment shown in panel B (81 cells were analyzed for each condition). (D) Control and JAM-A siRNA transfected MDCK cells were stained for GEF-H1. Junctional recruitment was quantified (n for control siRNA, 68; JAM-A siRNA, 63; shown are datapoints and means±1SD). (E) MDCK cells plated on Matrigel-coated glass coverslips or hydrogels were stained for JAM-A, occludin, and DNA to visualize nuclei. Shown are confocal sections taken at the junctional area and the lower half of the lateral membrane. Magnification bars, 20µm.
